# Fibronectin mediates activin A-promoted human trophoblast migration and acquisition of endothelial-like phenotype

**DOI:** 10.1186/s12964-023-01463-z

**Published:** 2024-01-23

**Authors:** Xiangxin Lan, Ling Guo, Cuiping Hu, Qian Zhang, Jianye Deng, Yufeng Wang, Zi-Jiang Chen, Junhao Yan, Yan Li

**Affiliations:** 1 Institute of Women, Children and Reproductive Health, Shandong University, Jinan, 250012 Shandong China; 2 State Key Laboratory of Reproductive Medicine and Offspring Health, Shandong University, Jinan, 250012 Shandong China; 3 Medical Integration and Practice Center, Shandong University, Jinan, 250012 Shandong China; 4 Research Unit of Gametogenesis and Health of ART-Offspring Chinese Academy of Medical Sciences (No. 2021RU001), Jinan, 250012 Shandong China

**Keywords:** Activin A, Fibronectin, Trophoblast, Migration, Endothelial-like tube formation, Pregnancy

## Abstract

**Background:**

During human early placentation, a proportion of extravillous trophoblasts (EVTs) migrate to the maternal decidua, differentiating into endovascular EVTs to remodel spiral arteries and ensure the establishment of blood circulation at the maternal-fetal interface. Inadequate EVT migration and endovascular differentiation are closely associated with adverse pregnancy outcomes such as miscarriage. Activin A and fibronectin are both secretory molecules abundantly expressed at the maternal-fetal interface. Activin A has been reported to regulate EVT biological functions. However, whether fibronectin mediates activin A-promoted EVT migration and acquisition of endothelial-like phenotype as well as the underlying molecular mechanisms remain unknown. Additionally, the role of fibronectin in pregnancy establishment and maintenance warrants further investigation.

**Methods:**

Primary and immortalized (HTR8/SVneo) human EVTs were used as in vitro study models. Cultured human first-trimester chorionic villous explants were utilized for ex vivo validation. A local fibronectin knockdown model in ICR mouse uteri, achieved by nonviral in vivo transfection with small interfering RNA (siRNA) targeting fibronectin 1 (si-*Fn1*), was employed to explore the roles of fibronectin in the establishment and maintenance of early pregnancy.

**Results:**

Our results showed that activin A treatment significantly induced fibronectin 1 (*FN1*) mRNA expression and fibronectin protein production, which is essential for human trophoblast migration and endothelial-like tube formation. Both basal and activin A-upregulated fibronectin expression were abolished by the TGF-β type I receptor inhibitor SB431542 or siRNA-mediated knockdown of activin receptor-like kinase (*ALK4*) or *SMAD4*. Moreover, activin A-increased trophoblast migration and endothelial-like tube formation were attenuated following the depletion of fibronectin. Fibronectin knockdown via intrauterine siRNA administration reduced CD31 and cytokeratin 8 (CK8) expression at the maternal-fetal interface, resulting in a decrease in the number of implantation sites and embryos.

**Conclusions:**

Our study demonstrates that activin A promotes trophoblast cell migration and acquisition of endothelial-like phenotype via ALK4-SMAD2/3-SMAD4-mediated fibronectin upregulation. Furthermore, through a local fibronectin knockdown model in mouse uteri, we found that the absence of fibronectin at the maternal-fetal interface impedes endovascular migration of trophoblasts and decidual vascularization, thereby interfering with early embryo implantation and the maintenance of pregnancy. These findings provide novel insights into placental development during early pregnancy establishment and contribute to the advancement of therapeutic approaches for managing pregnancy complications related to trophoblast dysfunction.

**Supplementary Information:**

The online version contains supplementary material available at 10.1186/s12964-023-01463-z.

## Introduction

Following human embryo implantation, extravillous trophoblasts migrate and invade the decidua via interstitial and endovascular routes for placental attachment and uterine spiral artery remodeling, respectively [1]. Insufficient EVT migration, invasion and vascular remodeling contribute to placental dysfunction, which is associated with infertility and various pregnancy complications, including miscarriage, preeclampsia, fetal growth restriction, and stillbirth [2–4].

The cellular behaviors of EVTs are regulated by a series of cytokines, growth factors, and cell signaling pathways [5–7]. Activin A, which belongs to the transforming growth factor-β (TGF-β) superfamily [8], was initially identified as an ovarian hormone that stimulates follicle stimulating hormone (FSH) release by the pituitary gland [9, 10]. Subsequent research has demonstrated the roles of activin A in decidualization, embryo implantation, placentation, and pregnancy maintenance. Lower activin A levels have been reported in the maternal serum of patients with ectopic pregnancies [11, 12] and miscarriage [13], even before the onset of clinical symptoms. Moreover, increased concentrations of activin A have been found in uterine washings from women who successfully became pregnant following intrauterine insemination [14], highlighting the essential role of activin A in pregnancy establishment and maintenance. Therefore, elucidating the underlying molecular mechanism of altered activin A levels in these complications holds promise for developing novel diagnostic and therapeutic regimens for infertility and pregnancy-related disorders.

Activin A signals by interacting with type I (ActRI or ALK4) and type II (ActRII) receptors [15]. The binding of activin A to its type II receptor recruits and phosphorylates type I receptors, forming phosphorylated heteropolymers. Then, the intracellular SMAD2/3 signaling pathway is triggered, forming complexes with SMAD4, which translocate into the nucleus and modulate the transcription of target genes [16]. Activin A can also activate non-SMAD pathways, such as the MAPK/ERK, PI3K/Akt, and JNK pathways [17–19]. Our group has previously demonstrated the pro-invasive role of activin A in trophoblast invasion [20, 21], as well as the role of activin A in promoting HTR8/SVneo cell endothelial-like tube formation by upregulating vascular endothelial growth factor-A (VEGF-A) expression [22]. However, the upregulation of VEGF-A could not fully explain activin A-promoted trophoblast migration and acquisition of endothelial-like phenotype, suggesting the involvement of other molecules in this complex regulatory network.

Fibronectin, a glycoprotein encoded by the *FN1* gene, is abundantly present in the extracellular matrix (ECM) and plays essential roles in cell adhesion, migration, inflammation, wound healing, and tissue integrity and remodeling [23, 24]. There are many studies on the role of upregulated fibronectin in tumor growth, angiogenesis, metastasis and progression [25, 26]. The role of fibronectin in placental trophoblasts attracted our attention given the similarities between tumors and the human placenta [27, 28]. Notably, among all human tissues and single cell types, fibronectin is most abundant in human placental tissue and extravillous trophoblasts [29, 30]. According to the research of Zhu et al. [31], transcriptome profiles of HTR8/SVneo trophoblast cells with and without activin A treatment show the most significant changes in the ECM interaction pathway. Moreover, in normal pregnancies, maternal circulating concentrations of both fibronectin [32, 33] and activin A [34, 35] tend to increase progressively throughout pregnancy.

In the present study, we validated our hypothesis that fibronectin participates in activin A-promoted trophoblast migration and endothelial-like tube formation through the ALK4-SMAD2/3-SMAD4 signaling pathway. By knocking down fibronectin in the uteri of ICR mice, we observed disorders in maternal-fetal interface vascularization and miscarriage phenotypes, mirroring the clinical occurrence of miscarriage in patients with low activin A and fibronectin levels [13, 36]. Our findings underscore *FN1* as a novel activin A target gene in human trophoblast cells, offering new insights into the signaling mechanisms and potential targets for the clinical management of infertility and pregnancy-related disorders related to trophoblast dysfunction.

## Materials and methods

### Culture of HTR8/SVneo immortalized human primary EVTs

The HTR8/SVneo immortalized human EVT cell line (ATCC CRL-3271, Manassas, VA, USA) was cultured in Dulbecco’s modified Eagle medium nutrient mixture F-12 Ham (DMEM/F12; Gibco) supplemented with 10% (vol/vol) fetal bovine serum (FBS; Gibco, Grand Island, NY, USA), 100 U/mL penicillin, and 100 μg/mL streptomycin (HyClone, Logan City, UT, USA). Cells were incubated in a 37 °C humidified incubator with 5% CO_2_, and the medium was changed every other day. To avoid possible interference from FBS, cells were treated with activin A after a starvation period of 24 hours (h) in DMEM/F12 containing 0.1% (vol/vol) FBS, 100 U/mL penicillin and 100 μg/mL streptomycin.

### Isolation and culture of human primary EVTs

The use of human primary EVTs was approved by the research ethics committee of the School of Medicine, Shandong University. Chorionic villous tissue samples were collected from artificial abortion-vacuum aspiration during first trimester pregnancy (6-8 weeks) and then rinsed with phosphate-buffered saline (PBS) several times to remove blood. The isolation approach and culture of human primary EVTs have been described previously [37]. Briefly, the clean villous tissues were dissected into 1-2 mm fragments and plated in complete medium (DMEM/F12 with 10% fetal bovine serum, 100 U/mL penicillin, and 100 μg/mL streptomycin) for 3-5 days at 37 °C in a humidified atmosphere of 5% CO_2_ in air. Subsequently, nonattached pieces were washed away, and adherent explants and migrated EVTs were cultured for an additional 10-14 days. Primary EVTs were finally separated from attached fragments by trypsinization, and their purity was confirmed by positive IF staining for human leukocyte antigen G (HLA-G) and cytokeratin 7 (CK7).

### Culture of human early pregnancy chorionic villous explants and the outgrowth assay

Growth factor-reduced Matrigel (Corning, 354230) was submerged in ice in a 4 °C refrigerator to thaw overnight and diluted 1:1 (vol/vol) with 0.1% FBS DMEM/F12 to a final concentration of 5 mg/mL. Five hundred microliters of diluted Matrigel was added to each well of a 6-well plate and incubated at 37 °C for 2 h to solidify. Chorionic villous tissues from the first trimester of pregnancy (6-8 weeks) were dissected into 1-2 mm fragments, suspended in a small amount of 10% FBS DMEM/F12, seeded on Matrigel-coated plates and incubated at 37 °C. The cell outgrowth from the edge of the villous fragments was observed under a microscope. The explants were treated with activin A and siRNA at the emergence of outgrowth cells. Explant outgrowth was observed and photographed after specific time intervals. Area of explant outgrowth and migration were measured by ImageJ and quantified by the formula: explant migration = (48 h area – 24 h area)/24 h area.

### RNA-sequencing (RNA-seq) data collection and analysis

The RNA-Seq data of HTR8/SVneo cells treated with activin A were downloaded from the Sequence Read Archive (SRA) database (PRJNA640458). We performed an analysis of differentially expressed genes (DEGs) in HTR8/SVneo cells after activin A exposure for 6 h and 24 h using the DESeq2 R package [38] and enhanced volcano R package [39]. Genes that were significantly differentially expressed (fold change > 2 and *P* value < 0.05) after both 6 h and 24 h of activin A treatment were screened by plotting a Venn diagram [40]. These screened activin A-related DEGs were subjected to Mfuzz time series cluster analysis [41, 42], which identifies potential time series patterns and clusters genes with similar patterns. We selected Cluster 1 of a total of 339 genes, including *FN1,* and analyzed their potential functional pathways through Gene Ontology (GO)-based functional enrichment and annotation using the clusterProfiler R package [43]. R packages were obtained from the website Bioconductor (http://www.bioconductor.org/), and the results were visualized with R studio [44].

### Antibodies and reagents

Anti-fibronectin rabbit polyclonal antibody (ab2413) and anti-CD31 rabbit recombinant multiclonal antibody (ab281583) were purchased from Abcam (Cambridge, MA); SMAD2 (L16D3) mouse monoclonal antibody (# 3103), phospho-SMAD2 (138D4) rabbit monoclonal antibody (# 3108), SMAD3 (C67H9) rabbit monoclonal antibody (# 9523), phospho-SMAD3 (C25A9) rabbit monoclonal antibody (# 9520), SMAD4 (D3M6U) rabbit monoclonal antibody (# 38454), horseradish peroxidase (HRP)-linked anti-mouse IgG (# 7076) and anti-rabbit IgG (# 7074) were purchased from Cell Signaling (Beverly, MA); mouse monoclonal antibody HLA-G was purchased from EXBIO (#11-499-C100**)**; cytokeratin 7-specific rabbit polyclonal antibody (# 17513-1-AP) was purchased from Proteintech (Wuhan, China); mouse monoclonal anti-α-Tubulin antibody (sc-23948) was obtained from Santa Cruz Biotechnology (Santa Cruz, CA); mouse monoclonal anti-cytokeratin 8 antibody (GB12233-100) was obtained from Servicebio (Wuhan, China); anti-Mouse Secondary Antibody, Alexa Fluor 488 (#A11001), anti-Rabbit Secondary Antibody, Alexa Fluor 594 (# A11012) and *ACVR1B* (*ALK4*) TaqMan primers (# 433182) were obtained from Thermo Fisher (Waltham, MA); recombinant human/mouse/rat activin A protein was provided by R&D (Minneapolis, MN); SB431542 (TGF-beta type I receptor inhibitor) (# S4317) was purchased from Sigma Aldrich (St. Louis, MO).

### Endothelial-like tube formation assay

Growth factor-reduced Matrigel (Corning, 354230) was submerged in ice in a 4 °C refrigerator to thaw overnight and diluted 1:1 (vol/vol) with 0.1% FBS DMEM/F12 to a final concentration of 5 mg/mL. Fifty microliters of diluted Matrigel was added to each well of a 96-well plate and incubated at 37 °C for 2 h to solidify. After pretreatment with either vehicle or activin A (50 ng/mL, a concentration comparable to physiopathological levels [45, 46] and effective in consistently upregulating the mRNA and protein levels of fibronectin), HTR8/SVneo cells were seeded onto Matrigel-coated plates (4.0 × 10^4^ cells suspended in 50 μL 0.1% FBS DMEM/F12 per well) and incubated at 37 °C for 12 h. Digital images were taken with a light microscope, and total tube length was quantified with ImageJ software.

### Wound healing assay

Pretreated cells were seeded in 6-well plates and cultured until the cells fully covered the bottom of the well. A wound was made with a scratch by a 200 μL sterile pipette tip in every group, and the cell debris was removed by washing with PBS. The remaining cells were further cultured, and the wound areas at 0, 12 and 24 h were measured. The wound closure rate was calculated by using the following formula: (wound area at 0 h-wound area at indicated time)/wound area at 0 h × 100%.

### Cell proliferation assay

The effect of siRNA targeting human *FN1* (si-*FN1*) and activin A on the proliferation of HTR8/SVneo cells was analyzed by cell counting kit-8 (CCK-8, Beyotime Institute of Biotechnology, China) and 5-ethynyl-2′-deoxyuridine (EdU) kit (Ribobio, China) according to the manufacturer’s instructions. In brief, for the CCK-8 assay, cultured HTR8/SVneo cells were suspended in DMEM/F12 with 0.1% FBS and incubated in a 96-well plate for the indicated durations. CCK-8 reagent (10 μL/well) was added to the culture medium, and the plate was incubated for 2 h at 37 °C. The cell proliferative potential was assessed by measuring the absorbance at 450 nm. For the EdU labeling assay, pretreated HTR8/SVneo cells were exposed to 50 μM EdU for 2 h. Subsequently, newly replicated DNA was stained with 1× Apollo reaction cocktail (red), and total DNA was stained with Hoechst 33342 (blue). Under a fluorescence microscope, the stained cells were photographed, and the rate of EdU-positive cells was used to evaluate the proliferation of cells.

### Reverse transcription quantitative real-time PCR (RT–qPCR)

Total RNA was extracted from cells with an RNA-Quick Purification Kit (Yishan Biotechnology, Shanghai, China) according to the manufacturer’s instructions. TRIzol reagent (Thermo Fisher) was used to isolate total RNA from uterine horns collected from mice on day 6 of pregnancy. cDNA was synthesized from 1 μg of total RNA using the Reverse Transcription Reagent Kit (Takara, Shiga, Japan). RNA expression was measured by RT–qPCR using the SYBR-Green method (Takara) based on the manufacturer’s instructions and conducted on the Roche LightCycle 480 (Roche, Penzberg, Germany). The mRNA level of *ALK4* was assayed by TaqMan gene expression assay (Applied Biosystems). The primers used were as follows: human glyceraldehyde-3-phosphate dehydrogenase (*GAPDH*), 5′-GAGTCAACGGATTTGGTCGT-3′ (forward) and 5′-GACAAGCTTCCCGTTCTCAG-3′ (reverse); *FN1*, 5′-ACAGAACTATGATGCCGACCAGAAG-3′ (forward) and 5′-CTGATCTCCAATGCGGTACATG-3′ (reverse); *SMAD4*, 5′-TGGCCCAGGATCAGTAGGT-3′ (forward) and 5′-CATCAACACCAATTCCAGCA-3′ (reverse). In addition, the primer sequences of *Fn1* and *Rpl7* for mice were as follows: mus-*Fn1*, 5′-AGTTTGTGCATGGTGTCCGA-3′ (forward) and 5′-CAGTTGTGCCTGGGTAGGTC-3′ (reverse); *Rpl7*, 5′-AGCTGGCAACTTCTATGTGC-3′ (forward) and 5′-CGCAGCATGTTAATTGAAGCC-3′ (reverse). For each sample, at least three independent experiments were performed in triplicate, and the 2^-ΔΔCq^ method was used for relative quantification of gene expression.

### Western blot

Cells and mouse uterine tissue were lysed in cell lysis buffer (Cell Signaling) and RIPA lysis buffer (Beyotime Institute of Biotechnology, p0013b), respectively, supplemented with a protease/phosphatase inhibitor cocktail (Cell Signaling). Extracts were centrifuged at 14000×g for 10 min at 4 °C, and a BCA protein assay kit (Thermo Fisher) was used to detect the supernatant protein concentrations. Equal amounts of protein (15 μg) were separated on an SDS–PAGE gel by electrophoresis and then electrotransferred to PVDF membranes. Membranes were blocked with 5% nonfat milk or 5% bovine serum albumin (BSA, Sigma) at room temperature for 1 h and then incubated with primary antibodies against α-Tubulin (1:1000), SMAD2 (1:1000), phospho-SMAD2 (1:1000), SMAD3 (1:1000), phospho-SMAD3 (1:2000), SMAD4 (1:1000) or fibronectin (1:1000) at 4 °C overnight. After incubation with appropriate HRP-conjugated secondary antibodies for 1 h at room temperature, signals were detected by Prolighting HRP agent (Thermo Fisher). Densitometric quantification was performed using Image-Pro Plus software with α-Tubulin, SMAD2 or SMAD3 for normalization.

### Small interfering RNA transfection

Trophoblast cell transfection was performed by using Lipofectamine RNAiMAX Transfection Reagent (Thermo Fisher) and Opti-MEM I (Gibco) according to the manufacturer’s instructions. Fifty percent confluent cells were transfected for 24 h with 20 nM ON-TARGETplus Nontargeting Control Pool (si-Ctrl) or ON-TARGETplus SMARTpool siRNAs targeting human *FN1*, *ALK4* or *SMAD4* (Dharmacon, GE Healthcare Life Sciences). In vivo delivery of ON-TARGETplus Mouse *Fn1* siRNA-SMARTpool or si-Ctrl in the mouse uterus was mediated by in vivo-jetPEI (Polyplus transfection®, New York, USA) according to the manufacturer’s instructions. In brief, 100 μL of in vivo-jetPEI/siRNA complex (N/P ratio = 8) was prepared with 10 μg siRNA and 1.6 μL in vivo-jetPEI in 5% glucose solution. Every mouse was injected with 10 μL of in vivo-jetPEI/siRNA complex on each side of the uterine horn. RT–qPCR or Western blot analysis was used to assess transfection efficiency.

### Immunocytochemistry

Human primary EVTs were cultured in glass bottom dishes (NEST.801002). After fixation with 4% paraformaldehyde (PFA), primary EVTs were permeabilized with 0.3% Triton X-100 and blocked with 10% goat serum (ASGB-BIO, ZLI-9056) for 1 h. Then, the cells were incubated with primary antibodies against CK7 (1:200), HLA-G (1:100) or fibronectin (1:300) overnight at 4 °C. Positive staining of CK7 and fibronectin was detected using a fluorescent goat anti-rabbit secondary antibody (1:800), positive staining of HLA-G was detected using a fluorescent goat anti-mouse secondary antibody (1:800) and nuclei were counterstained with Hoechst. Images were captured under a confocal laser scanning microscope.

### Animal experiments

All mice used in our experiments were wild-type ICR mice, which were housed in a temperature-controlled room under a constant 12 h/12 h light/dark cycle in the Experimental Animal Center of Shandong University (EAC-SDU, Jinan, China) according to NIH and institutional guidelines for the use and care of laboratory animals. Female mice (8-12 weeks of age) were mated overnight, and the day when a vaginal plug was observed was designated day 1 (D1) of pregnancy. The pregnant mice underwent surgery through a small abdominal incision after anesthesia on D4 to inject the in vivo-jetPEI/si-*Fn1* complex or in vivo-jetPEI/si-Ctrl complex into the bilateral uterine horns. In this way, we obtained mice with localized fibronectin knockdown in the uterus (si-*Fn1* group) before embryo implantation and their controls (NC group). On D6, we used isoflurane to anesthetize the mice and injected Chicago Blue dye solution (150 μL/mouse) intravenously to visualize the implantation sites (IS). Uterine tissues were collected for subsequent PCR and Western blot. On D8, the mice were sacrificed by cervical dislocation, and the morphology and number of embryos were recorded. The implantation segments were separated for further immunohistochemistry and immunofluorescence. At least three mice from each group were used for individual experiments.

### Immunohistochemistry (IHC)

The uterine tissues of mice were fixed in 4% PFA, dehydrated sequentially in a concentration gradient of ethanol, cleared in xylene and embedded in paraffin wax. Tissue samples from at least 3-5 mice per group were sectioned at a thickness of 5 μm, and slides from the center of the uterus were used for IHC, which was performed by using the Rabbit Two-Step Assay Kit (ZSGB-BIO, PV-9001) according to the manufacturer’s instructions. The primary antibody used was CD31 (1:4000; Abcam).

### Immunofluorescence (IF)

Paraffin-embedded sections of mouse D8 uterus underwent dewaxing, rehydration and antigen retrieval (EDTA buffer pH 9.0). Then, the sections were permeabilized with 0.3% Triton X-100 and blocked with 10% goat serum for 1 h prior to CK8 (1:1000) and CD31 (1:100) overnight at 4 °C. Fluorescence-conjugated Alexa Fluor secondary antibodies were used (1:800), and sections were mounted on slides with mounting medium with DAPI (Abcam, ab104139). Images were captured under a confocal laser scanning microscope.

### Statistical analysis

All results are presented as the mean ± SEM of at least three independent experiments. The normality of the data was determined by the Shapiro–Wilk test and Q–Q plot. Normally distributed data were analyzed using the Student’s t test for two groups or analysis of variance (ANOVA) for three or more groups; non-normally distributed data were analyzed by the Mann–Whitney U test. Data comparisons were performed using SPSS 26.0 and PRISM software (GraphPad Software, Inc.). *P* < 0.05 was considered to be statistically significant.

## Results

### Activin a upregulates fibronectin expression in HTR8/SVneo cells

**Fig. 1:**
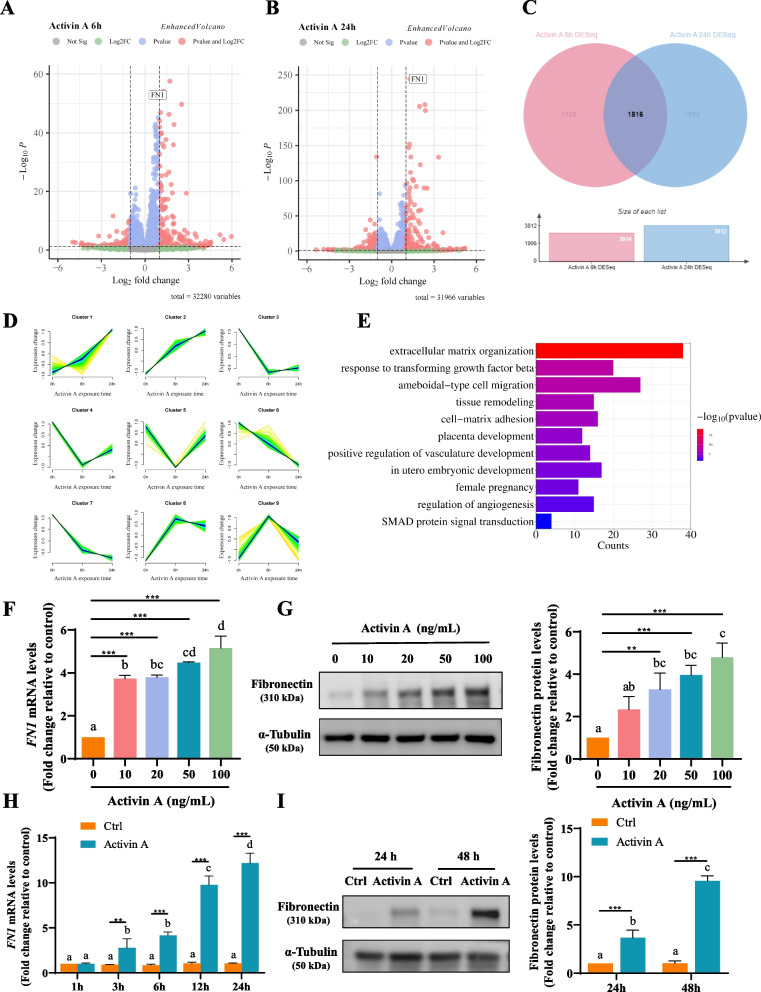
Activin A increases fibronectin expression in HTR8/SVneo cells. **A** and **B**, Volcano plots obtained from RNA-Seq analysis of HTR8/SVneo cells treated with or without activin A for 6 h (**A**) or 24 h (**B**). **C**, The Venn diagram displays an overlap of 1816 DEGs between 6 h and 24 h of activin A treatment. **D**, The Mfuzz time series clustering of 1816 DEGs after 0-6-24 h of activin A treatment. The color gradient, ranging from yellow to navy blue, signifies the increasing suitability of gene trends in accordance with the alterations within the cluster. A total of 339 genes, including *FN1*, were in Cluster 1. **E**, GO enrichment analysis of Cluster 1. **F** and **G**, HTR8/SVneo cells were treated with different concentrations (0, 10, 20, 50 or 100 ng/mL) of activin A for 24 h, and the mRNA (**F**) and protein (**G**) levels of fibronectin were examined by RT–qPCR and Western blot analysis, respectively. **H** and **I**, HTR8/SVneo cells were treated with vehicle (Ctrl) or 50 ng/mL activin A for different lengths of time (1, 3, 6, 12 or 24 h for mRNA; 24 or 48 h for protein), and the mRNA (**H**) and protein (**I**) levels of fibronectin were examined by RT–qPCR and Western blot analysis, respectively. Quantitative results are expressed as the mean ± SEM of at least three independent experiments. One-way ANOVA was used for analyses of the five groups in (**F**) and (**G**). Two-way ANOVA was used for grouped analyses in (**H**) and (**I**). Groups without letters in common are significantly different from each other (*P* < 0.05). *P* values of primary interest are denoted as * < 0.05, ** < 0.01, and *** < 0.001

To explore the molecular mechanism by which activin A promotes trophoblast migration and acquisition of endothelial-like phenotype, we analyzed DEGs in HTR8/SVneo cells treated with or without activin A (50 ng/mL) for 6 or 24 h by RNA-seq analysis. The volcano plots (Fig. 1A and B) show that *FN1* was upregulated following activin A treatment at both 6 and 24 h. Employing a Venn diagram (Fig. 1C), we discerned 1816 activin A-related DEGs at both 6 and 24 h and further classified them into 9 clusters, as shown in Fig. 1D, utilizing the Mfuzz R package tool. Within cluster 1, the expression of *FN1* and an additional 338 genes (including *SOX4*, *TGFB1*, *IGFBP4*, *JAG1*, *ITGA5*, *MMP2*, *HIF1A*, *BMP1*, *CD37*, *ITGB1*, *LIF*, *FOXC2,* etc., Figure 1**–**Source Data 3. Excel file for Figure 1D) increased remarkably from 0 to 6 and to 24 h of activin A exposure (Fig. 1D). Functional enrichment and GO analysis of these 339 genes revealed that activin A treatment significantly altered the transcription of genes associated with extracellular matrix organization, as ranked by both the -log10 (*P* value) and gene counts. Other biological processes that are significantly enriched are ameboidal-type cell migration, tissue remodeling, cell-matrix adhesion, positive regulation of vasculature development, and regulation of angiogenesis, all of which are related to important biological functions of trophoblasts during placental development and embryonic development in utero. SMAD protein signal transduction after activin A treatment was also enriched (Fig. 1E). Extracellular matrix components are important for placental tissue integrity [47]. Matrix assembly is usually initiated by ECM glycoproteins that bind to cell surface receptors, such as fibronectin dimers that bind to α5β1 integrins [48]. This brings our focus to *FN1*, which encodes fibronectin, a glycoprotein involved in angiogenesis, cell adhesion and migration [49–51]. To validate the RNA-Seq findings, we examined the effects of activin A on fibronectin expression in human trophoblast cells by treating HTR8/SVneo cells with activin A at different dosages and for different durations. RT–qPCR and Western blot analysis revealed that activin A significantly upregulated fibronectin mRNA and protein levels in HTR-8/SVneo cells in a dose-dependent (Fig. 1F and G) and time-dependent (Fig. 1H and I) manner.

### Activin A increases HTR8/SVneo cell migration and endothelial-like tube formation by upregulating fibronectin expression

**Fig. 2:**
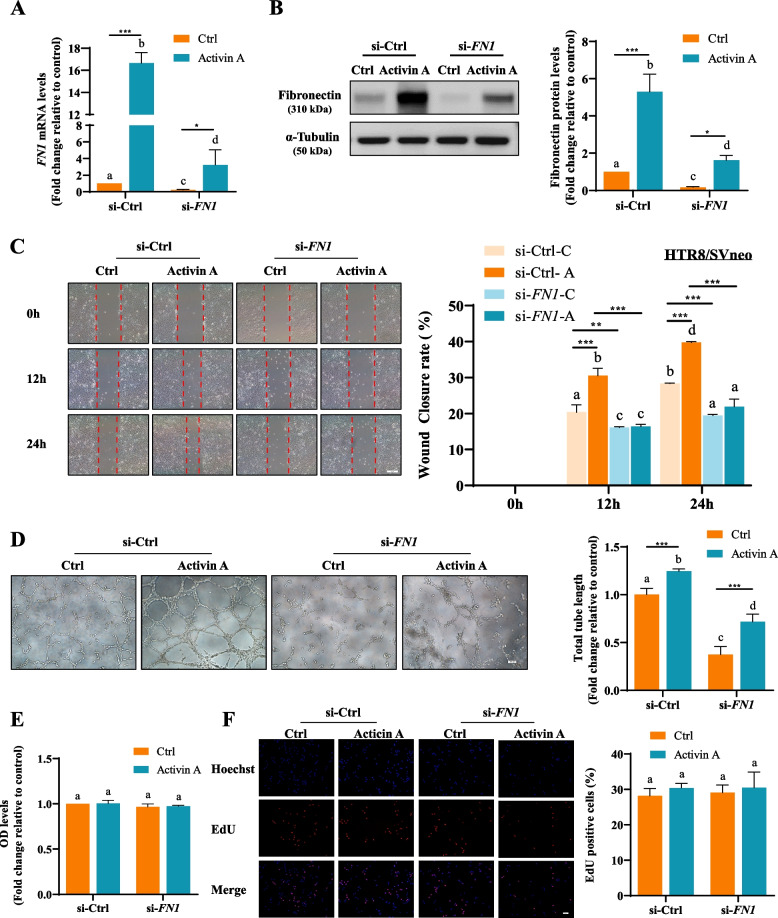
Fibronectin is involved in activin A-induced cell migration and endothelial-like tube formation in HTR8/SVneo cells. **A** and **B**, HTR8/SVneo cells were transfected for 48 h with 20 nM ON-TARGETplus Non-targeting Control Pool (si-Ctrl) or ON-TARGETplus SMARTpool siRNAs targeting human *FN1* (si-*FN1*) prior to treatment with Ctrl or 50 ng/mL activin A for an additional 24 h. The expression levels of *FN1* mRNA (**A**) and fibronectin protein (**B**) were examined by RT–qPCR and Western blot. **C**, HTR8/SVneo cell migration was examined by wound healing assay. Representative diagrams of wound healing assays were taken at 0, 12 and 24 h. **D**, Effects of *FN1* siRNA treatment on activin A-induced HTR8/SVneo cell endothelial-like tube formation were examined after transfection for 48 h with si-Ctrl or si-*FN1* with or without activin A for 12 h. Left panels show representative images from the endothelial-like tube formation assays. The right panel shows summarized quantitative results from image analysis of total tube length. **E** and **F**, HTR8/SVneo cells were given the same interventions as in **A** and **B**, and cell proliferation was detected by CCK-8 (**E**) and EdU (**F**) at 24 h. The results are presented as the mean ± SEM of three independent experiments. ANOVA was used for quantitative grouped analyses. Columns without letters in common are significantly different (*P* < 0.05). *P* values of primary interest are denoted as * < 0.05, ** < 0.01, and *** < 0.001

Our previous study demonstrated that activin A significantly enhanced HTR8/SVneo cell endothelial-like tube formation [22]. Tamminen JA et al. reported a reduction in mesothelioma cell migration following the inhibition of activin A activity [52]. To elucidate the functional roles of upregulated fibronectin after activin A treatment, we transfected HTR8/SVneo cells with si-*FN1* prior to 50 ng/mL (comparable to physiological concentration) activin A treatment. As shown in Fig. 2, si-*FN1* significantly decreased fibronectin mRNA and protein levels in both basal and activin A-treated HTR8/SVneo cells (Fig. 2A and B). Increased migration ability, as assessed by a wound healing assay, was observed in HTR8/SVneo cells after activin A treatment, while fibronectin knockdown abolished activin A-promoted cell migration (Fig. 2C). As assessed by matrigel endothelial-like tube formation assays, acquisition of endothelial-like phenotype was significantly increased after activin A treatment, while transfection of si-*FN1* significantly attenuated both basal and activin A-promoted HTR8/SVneo cell endothelial-like tube formation (Fig. 2D). Furthermore, the CCK-8 assay (Fig. 2E) and EdU labeling assay (Fig. 2F) did not show any significant effect of activin A or fibronectin knockdown on the cell proliferation rate of HTR8/SVneo cells (*P* > 0.05). These results suggest that fibronectin mediates both basal and activin A-promoted HTR-8/SVeno cell migration and endothelial-like tube formation.

### Activin A upregulates fibronectin expression through ALK4-activated SMAD2/3-SMAD4 signaling in HTR8/SVneo cells

**Fig. 3:**
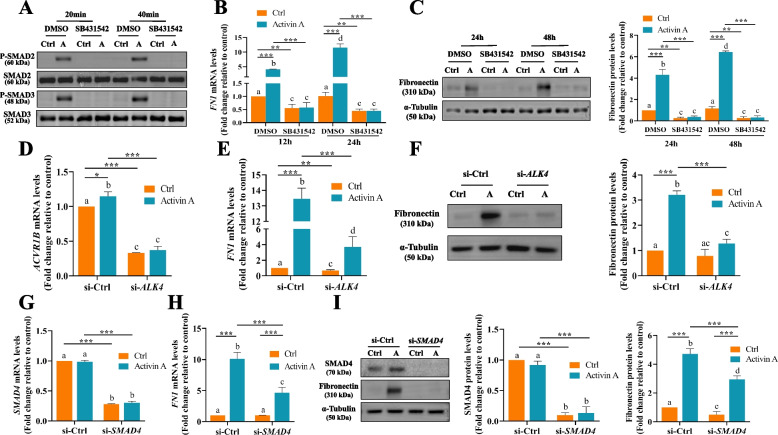
Activin A increases fibronectin expression through ALK4-activated SMAD2/3-SMAD4 signaling in HTR8/SVneo cells. **A**, **B** and **C**, HTR8/SVneo cells were pretreated for 1 h with vehicle control dimethylsulfoxide (DMSO) or 10 μm SB431542 before treatment without (Ctrl) or with 50 ng/mL activin A for 20 and 40 minutes for P-SMAD2/SMAD2 and P-SMAD3/SMAD3 protein (**A**), 12 and 24 h for *FN1* mRNA (**B**) or 24 and 48 h for fibronectin protein (**C**), examined by RT–qPCR or Western blot, respectively. **D**, **E**, and **F**, HTR8/SVneo cells were treated with or without 50 ng/mL activin A after transfection with 20 nM si-Ctrl or si-*ALK4*. The mRNA level of *ALK4 (ACVR1B)* was examined by TaqMan gene expression assay (**D**). The mRNA and protein levels of fibronectin were measured using RT–qPCR (**E**) and Western blot (**F**), respectively. **G**, **H** and **I**, HTR8/SVneo cells were treated with or without 50 ng/mL activin A after transfection with 20 nM si-Ctrl or si-*SMAD4*. The mRNA and protein levels of SMAD4 and fibronectin were measured by RT–qPCR (**G** and **H**) and Western blot (**I**), respectively. Quantitative results are expressed as the mean ± SEM of at least three independent experiments. ANOVA was used for grouped analyses. Groups without letters in common are significantly different from each other (*P* < 0.05). *P* values of primary interest are denoted as * < 0.05, ** < 0.01, and *** < 0.001

To explore the involvement of the canonical SMAD-dependent signaling pathway in activin A-upregulated fibronectin expression, experiments using SB431542 (activin/TGF-type 1 receptor [ALK4/5/7] inhibitor), siRNA targeting *ALK4* (si-*ALK4*), and siRNA targeting *SMAD4* (si-*SMAD4*) were performed. First, HTR8/SVneo cells were pretreated with either vehicle control dimethylsulfoxide (DMSO) or 10 μm SB431542 for 1 h before 50 ng/mL activin A treatment. Western blot analysis revealed that 20 or 40 minutes of activin A treatment induced the phosphorylation of SMAD2/3, which was blocked by SB431542 pretreatment (Fig. 3A). Assessment of fibronectin mRNA and protein levels indicated that SB431542 pretreatment completely abolished the upregulation effect of activin A on fibronectin expression (Fig. 3B and C). Second, HTR8/SVneo cells were treated with activin A after siRNA-mediated depletion of *ALK4*. The results showed that si-*ALK4* significantly decreased *ALK4* mRNA levels (Fig. 3D) and attenuated activin A-upregulated fibronectin mRNA (Fig. 3E) and protein (Fig. 3F) levels in HTR8/SVneo cells. Third, siRNA-mediated SMAD4 knockdown significantly decreased SMAD4 expression levels (Fig. 3G and I) and attenuated activin A-upregulated fibronectin expression (Fig. 3H and I).

### Activin A increases human primary EVT migration and human villous explant outgrowth by upregulating fibronectin expression

**Fig. 4:**
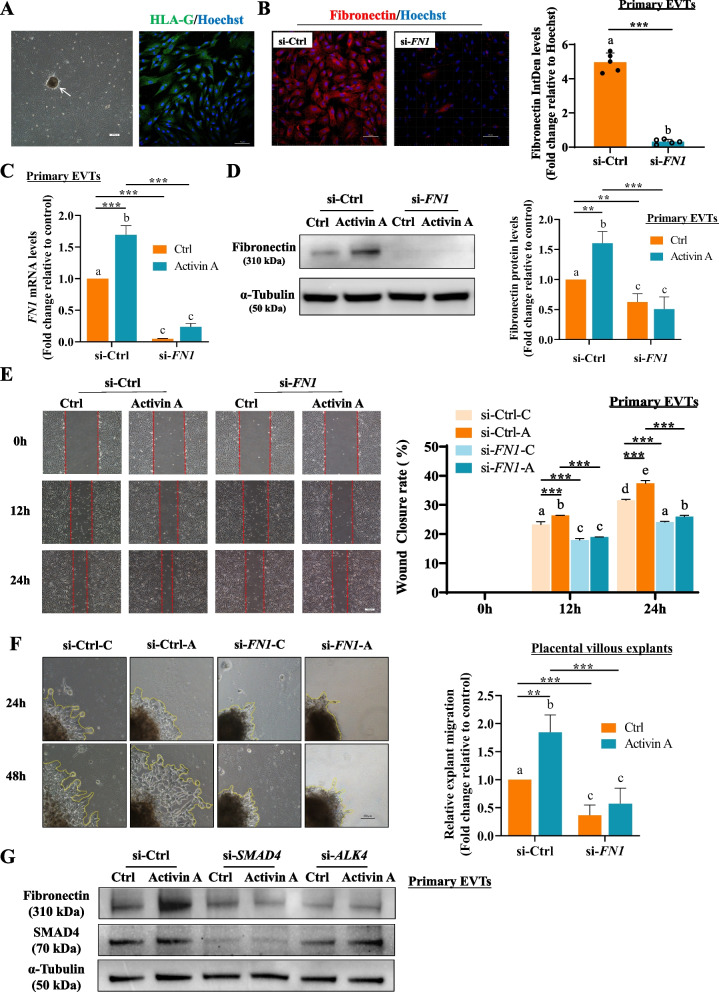
Activin A increases human primary EVT migration and human villous explant outgrowth by upregulating fibronectin expression through ALK4-SMAD4 signaling. **A**, Cellular morphology of primary EVT outgrowth (left panel, white arrow indicates the fragment of villous tissue) and purity of the primary human EVTs tested by IF staining for HLA-G (right panel). **B**, IF staining for fibronectin in human primary EVTs after transfection for 48 h with 20 nM si-Ctrl or si-*FN1*. The knockdown efficiency of fibronectin was evaluated by quantification of fibronectin IF integrated density (IntDen) normalized to Hoechst IF IntDen using ImageJ software. **C** and **D**, Human primary EVTs were transfected for 48 h with 20 nM si-Ctrl or si-*FN1* prior to treatment with Ctrl or 50 ng/mL activin A for an additional 24 h. The expression levels of *FN1* mRNA (**C**) and fibronectin protein (**D**) were examined by RT–qPCR and Western blot. **E**, Human primary EVT migration was examined by wound healing assay. Representative photographs of wound healing assays were taken at 0, 12 and 24 h. **F**, An ex vivo extravillous explant culture model was employed to further explore the role of activin A and fibronectin in the function of the human first trimester placenta. The placental villous explants were transfected with si-Ctrl or si-*FN1* after 24 h of culture, followed by Ctrl or 50 ng/mL activin A for an additional 24 h. Representative images were taken at 24 and 48 h of culture, and the right panel shows summarized quantitative results from image analysis of the explant outgrowth area. **G**, Human primary EVTs were transfected with 20 nM si-Ctrl, 20 nM si-*ALK4*, or 20 nM si-*SMAD4* and then treated with a vehicle control or 50 ng/mL activin A for an additional 24 h. Protein levels of SMAD4 and fibronectin were measured by Western blot. The results are presented as the mean ± SEM of three independent experiments. The *P* value in (**B**) was calculated by two-tailed Student’s t test. ANOVA was used for grouped analyses in (**C**)-(**F**). Groups without letters in common are significantly different from each other (*P* < 0.05). *P* values of primary interest are denoted as * < 0.05, ** < 0.01, and *** < 0.001

To validate our in vitro findings regarding the mediating role of fibronectin in activin A-promoted trophoblast migration, ex vivo experiments using first-trimester placental villous (6-8 weeks of gestation) fragments and primary cultures of human EVTs were performed. The purity of primary EVTs was examined using IF staining for the EVT-specific marker HLA-G (Fig. 4A) and trophoblast biomarker CK7 (Figure 4-Source data 1). IF staining for fibronectin was performed in primary EVTs that were outgrown from explants after si-Ctrl or si-*FN1* transfection to assess the knockdown efficiency of fibronectin (Fig. 4B). Consistent with the findings observed in HTR8/SVneo cells, activin A treatment significantly increased fibronectin mRNA and protein levels, which could be abolished by si-*FN1* transfection in human primary EVTs (Fig. 4C and D). Meanwhile, wound healing assays demonstrated that activin A treatment increased the migratory ability of human primary EVTs, similar to that observed in HTR8/SVneo cells, while fibronectin knockdown suppressed both basal and activin A-increased primary EVT migration (Fig. 4E). In addition, first-trimester placental explants were transfected with si-*FN1* prior to 50 ng/mL activin A treatment, and the area of outgrowth was recorded after the culture of explants for 24 and 48 h. The results showed that actinin A significantly increased primary EVT migration and outgrowth sprouting from the villous tips of explants, while fibronectin knockdown decreased the migration area of explants (Fig. 4F). To further confirm the role of SMAD-dependent signaling in activin A-upregulated fibronectin expression, human primary EVTs were transfected with si-*ALK4* and si-*SMAD4*, and Western blot analysis showed that knockdown of ALK4 or SMAD4 attenuated activin A-upregulated fibronectin expression (Fig. 4G). These findings further support that the upregulatory effects of activin A on fibronectin expression are mediated by ALK4-activated SMAD2/3-SMAD4 signaling in human trophoblast cells.

### Localized fibronectin knockdown in mouse uteri impairs vascular remodeling at the maternal-fetal interface and reduces implantation sites and embryo numbers

**Fig. 5:**
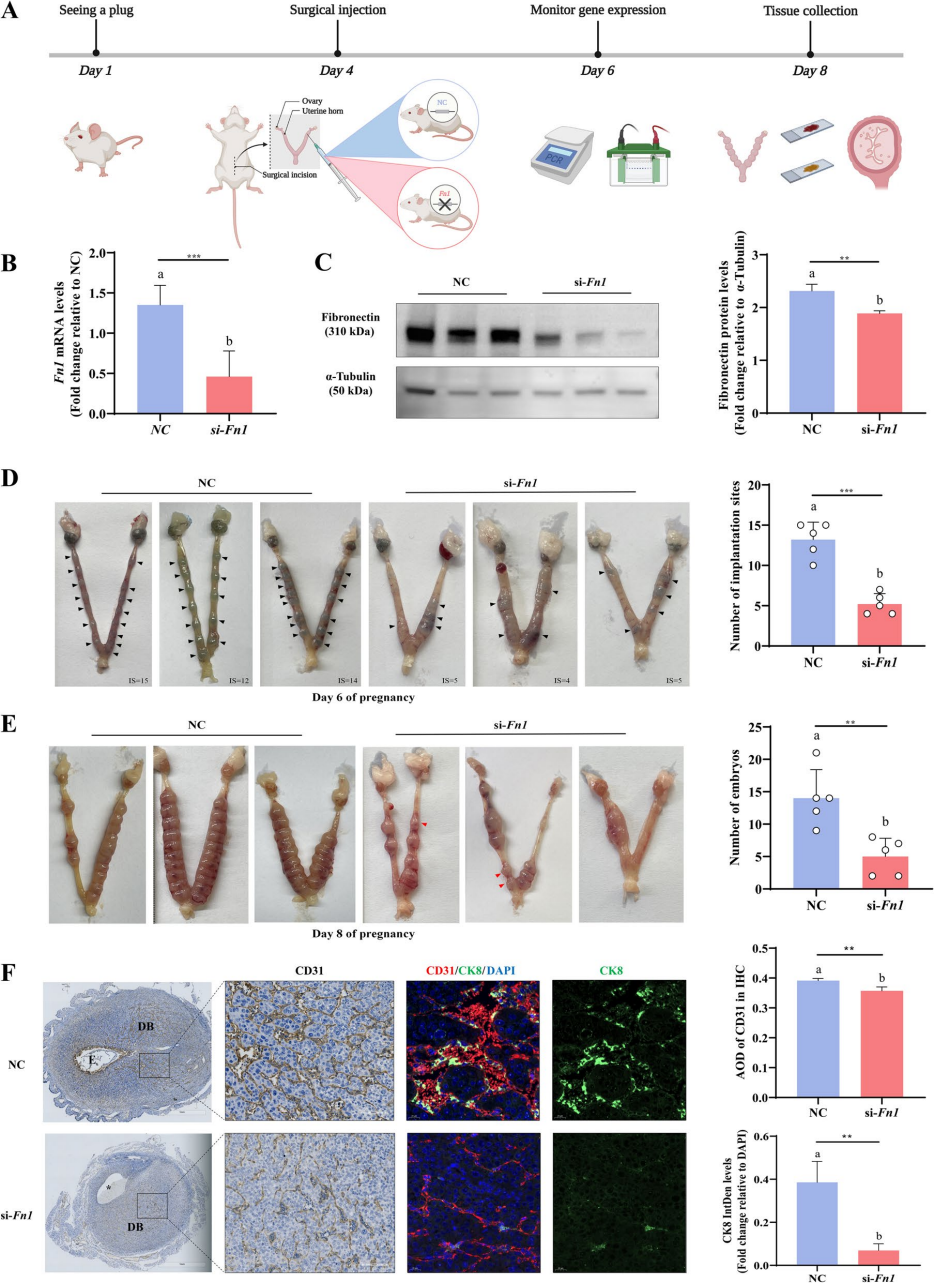
Localized knockdown of fibronectin in the uterine horns of mice decreased the number of implantation sites, the number of embryos successfully conceived, and the expression of CD31 and CK8. **A**, Treatment process of the mice is shown. The male and female mice were mated. The day when a vaginal plug was observed was designated day 1 (D1) of pregnancy. Before embryo implantation (morning of D4), the in vivo-jetPEI/si-*Fn1* complex or in vivo-jetPEI/si-Ctrl complex was injected as shown in (**A**). Then, the number of uterine implantation sites was counted on D6 of pregnancy (**D**, *n* = 5 mice/group), and uterine tissues were collected for PCR and Western blot to verify the knockdown efficiency of fibronectin (**B** and **C**, *n* = 3-6 mice/group). On D8, the number of successfully implanted embryos was counted (**E**, n = 5 mice/group), and the uterine sections were stained for CD31 and CK8. The average optical density (AOD) of the IHC staining intensity of CD31 was calculated by dividing integral optical density (IOD) by area. Fluorescence intensity of CK8 was quantified by dividing IntDen of CK8 by IntDen of DAPI. All images were quantitatively measured using ImageJ software (**F**, *n* = 4-6 mice/group). Black arrowheads indicate implantation sites; red arrowheads indicate resorption sites; E, embryo; asterisk, absence of embryo; DB, decidua basalis. The results are presented as the mean ± SEM from at least three independent experiments. *P* values were calculated by two-tailed Student’s t test or Mann–Whitney U test for the summarized quantitative results. Groups without letters in common are significantly different from each other (*P* < 0.05). *P* values of primary interest are denoted as * < 0.05, ** < 0.01, and *** < 0.001

Studies have shown that fibronectin concentrations in maternal circulation gradually increase during pregnancy [32, 33], and its expression in chorionic villous tissues is significantly downregulated in patients with spontaneous miscarriage [36]. In light of our in vitro results indicating that downregulation of fibronectin hampers human trophoblast cell migration and acquisition of endothelial-like phenotype, we sought to further understand the in vivo role of fibronectin in the maternal-fetal interface during early gestation. We used wild-type ICR mice to create a uterine fibronectin knockdown group (si-*Fn1*) and a control group (NC) by surgical injection of in vivo-jetPEI/si-*Fn1* complex or in vivo-jetPEI/si-Ctrl complex into the bilateral uterine horns on the morning of pregnancy day 4 (Fig. 5A). On day 6, successful fibronectin knockdown was confirmed using PCR and Western blot (Fig. 5B and C). We examined the implantation sites on day 6 by intravenous injection of Chicago blue dye solution. The blue staining of IS in the uterus after fibronectin knockdown was more diffuse, accompanied by localized uterine tissue edema. Additionally, the blue reaction indicated a significant decrease in the number of IS in the si-*Fn1* group compared to the NC group (Fig. 5D). On day 8, we observed a significant reduction in the number of embryos in the si-*Fn1* group compared to the control group (Fig. 5E). Several embryos in the si-*Fn1* group appeared to have smaller sizes, which we attributed to embryo resorption that occurred after embryo implantation. IHC revealed that the expression of the endothelial marker CD31 at the utero-maternal interface was significantly lower in the si-*Fn1* group than in the control group (Fig. 5F). In addition, we performed IF co-staining of CD31 with the trophoblast marker CK8 in sections from D8 pregnancy uteri. Consistent with the IHC results, the fluorescence intensity of CD31 was weaker in the uterus of mice in the si-*Fn1* group, along with noticeably thinner blood vessels, suggesting compromised decidual vascularization in the si-*Fn1* mice. This aligns with fewer CK8-positive endothelial EVTs in decidual blood vessels in the si-*Fn1* group compared to the NC group (Fig. 5F), indicating that fibronectin knockdown in the mouse uterus impairs trophoblast endovascular migration and decidual vascularization, ultimately affecting embryo implantation and pregnancy maintenance.

## Discussion

**Fig. 6:**
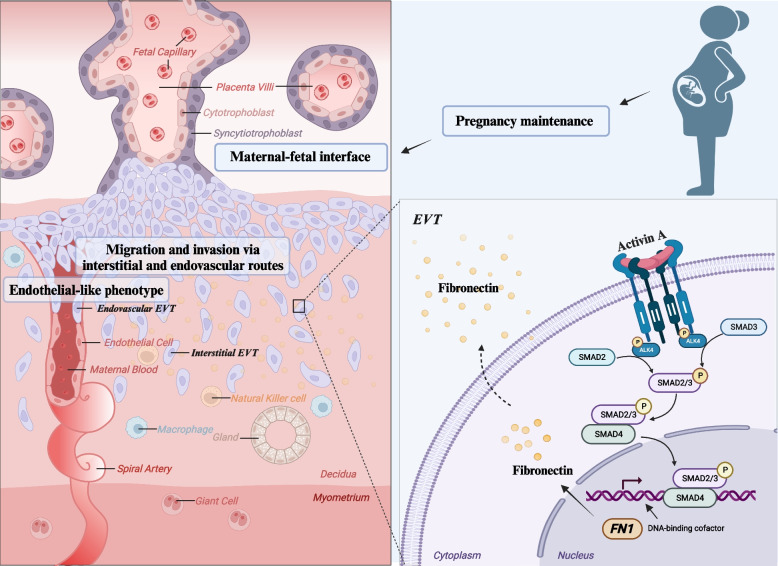
A signaling diagram illustrating the mediation role of fibronectin in activin A-increased human EVT migration, acquisition of endothelial-like phenotype and decidual vascularization at maternal-fetal interface. Activin A binds to the extracellular ligand-binding domain on trophoblast cells, and then, activin type I receptor (ActRI, ALK4) is recruited and phosphorylated. Phosphorylation of ALK4 leads to recruitment and phosphorylation of SMAD2/3, which forms a heterotrimeric SMAD complex with SMAD4. The trimeric SMAD complexes translocate from the cytoplasm to the nucleus and serve as transcription factors to modulate the expression of fibronectin. Fibronectin secreted and synthesized by EVTs is freely available in the microenvironment of the maternal-fetal interface and participates in activin A-promoted trophoblast migration and acquisition of endothelial-like phenotype, thus playing an important role in decidual vascularization and pregnancy maintenance. Created with BioRender.com

The present study shows the regulatory role of activin A in human trophoblast migration and acquisition of endothelial-like phenotype, mediated by an increase in fibronectin expression through the ALK4-SMAD2/3-SMAD4 signaling pathway, as presented in our summarized signaling diagram (Fig. 6). Moreover, we constructed an effective intrauterine fibronectin knockdown mouse model using an in vivo-jetPEI carrier of si-*Fn1* and further elucidated the role of fibronectin in early pregnancy establishment and vascular remodeling at the maternal-fetal interface. By elucidating the interaction pathways and functional mechanisms of activin A and fibronectin at the maternal-fetal interface in early pregnancy from in vitro, ex vivo and in vivo perspectives, these findings provide novel insights into the role of fibronectin in endovascular migration of trophoblast, decidual vascularization, and pregnancy maintenance.

Members of the TGF-β superfamily are known to play essential roles in early pregnancy, particularly in placental development and trophoblast function [53, 54]. Bone morphogenetic protein 4 induces the differentiation of human embryonic stem cells into trophoblasts [55], while TGF-β signaling regulates the differentiation program of EVTs, which migrate and invade the maternal decidua and its vasculature [53, 56]. Several studies in different trophoblast experimental models revealed TGF-β as a vital regulator of EVT biological behaviors, including invasion, migration, and acquisition of endothelial-like phenotype [57–59].

Activin A plays important roles in regulating the biological behavior of trophoblasts. Previous studies have shown that activin A promotes human trophoblast invasion, as evidenced by a Transwell invasion assay in HTR8/SVneo cells [20, 31, 60]. The vascular channels of human utero-placental bed are lined by trophoblasts, which acquire the functional characteristics of vascular endothelial cells [61]. Endovascular EVT invasion occurs with trophoblast cells migrating against the direction of blood flow at distal openings of uterine spiral arteries [62]. In this study, we assessed the effect of activin A on trophoblast cell migration using a wound healing assay in HTR8/SVneo cells and primary EVTs. The pro-migratory effect of activin A aligns with findings from two other studies involving Schwann cells and colorectal cancer cell lines [63, 64]. Furthermore, we utilized the intrinsic propensity of HTR8/SVneo cells for endothelial-like tube formation when cultured on Matrigel to investigate the underlying mechanisms at both the cellular and molecular levels [65]. These results corroborate another study, which confirmed activin A’s role in promoting endothelial-like tube formation in human trophoblasts by upregulating VEGF-A production [22]. Given the ethical restrictions, conducting interventional mechanistic studies in humans during pregnancy is unfeasible. The extravillous explant culture model employed in our study can partially simulate the process of intrauterine implantation and outgrowth of villi tissue in early pregnancy. However, the limitation of this method is that the explant outgrowth assay can only assess EVT outgrowth sprouting from the villous tips from a two-dimensional planar perspective, ignoring the three-dimensional interactions with the matrix and neighboring cells. Further research using organoids with 3D cocultures of different cell types could provide a more comprehensive understanding of EVT endovascular differentiation and the interplay of multiple responsible factors in the maternal-fetal interface microenvironment.

Altered fibronectin expression has been reported to be associated with numerous physiological and pathological processes, including tumor progression, hemostasis and thrombosis, wound healing and inflammation [66–68]. The regulation of fibronectin synthesis by activin A has also been reported in liver injury [69] and uterine leiomyoma [70]. In line with the ALK4-SMAD2/3-SMAD4 signaling pathway corroborated in this study, the role of activin A in leiomyoma cells is thought to be mediated by activation of the SMAD2/3 signaling pathway [70, 71]. Future studies that perform RNA sequencing on primary EVTs isolated from first-trimester placentas could more effectively validate our finding in HTR8/SVneo cells. In pregnant women, fibronectin acts as a “biological glue” at the chorion-decidua interface, serving as a marker for impending labor [72]. Previous studies have suggested that activin A, a secretory protein present in both the maternal and fetal circulations, undergoes significant changes throughout gestation [73], indicating its pivotal role in maintaining maternal-fetal homeostasis. Our findings in human trophoblast cells and chorionic villous explants assert that activin A-induced fibronectin upregulation promotes trophoblast migration and acquisition of endothelial-like phenotype, which is essential for vascular remodeling during embryo implantation.

Most previous studies investigating the role of fibronectin in vivo highly relied on the fibronectin gene knockout (*Fn*−/−) mouse model, which indicated embryonic level fatality [74]. To investigate the specific role of fibronectin in placentation and the underlying mechanism in vivo, we generated a localized fibronectin knockdown model in the uteri of ICR mice via nonviral transfection. The nonviral in vivo nucleic acid delivery method mediated by in vivo-jetPEI avoids the possible immune and toxic reactions caused by viral vectors, which remains a great challenge in clinical vaccine development and gene therapy applications [75, 76]. In the D8 si-*Fn1* uteri, CD31 and CK8 were both downregulated in our study. This finding contrasts with the study by Sung et al., which reported an increased percentage of endothelial cells alongside a decreased percentage of trophoblasts in the uterus of D13 mice with impaired spiral artery remodeling [77]. The reason for the inconsistency may be due, on the one hand, to the fact that our study focused on an earlier pregnancy stage when vascular remodeling is not yet completed [78]. On the other hand, fibronectin is a component of the ECM that supports the structural and functional integrity of blood vessels. In the spiral artery, cells including endothelial cells are embedded within the ECM [79]. Therefore, si-*Fn1* may not only interfere endovascular trophoblast migration but also compromise the integrity of decidual blood vessels. Although intrauterine administration of si-*Fn1* influences the entire uterine microenvironment, making it challenging to distinguish fibronectin’s roles in the endometrium versus embryonic trophoblasts, our findings help to affirm the important role of the secretory protein fibronectin in pregnancy maintenance and maternal-fetal interface vascularization during early pregnancy, thus providing candidate therapeutic targets for placental trophoblast dysregulation-related pregnancy complications.

## Conclusions

In this research, we pioneered the discovery that fibronectin mediates activin A-promoted trophoblast migration and acquisition of endothelial-like phenotype. Furthermore, we revealed that activin A-upregulated fibronectin expression is dependent on the activation of the ALK4-SMAD2/3-SMAD4 signaling pathway. Fibronectin at the maternal-fetal interface plays essential roles in endovascular trophoblast migration, decidual vascularization and pregnancy maintenance. These findings yield significant insights into placental development and contribute to the advancement of therapeutic approaches for managing pregnancy complications associated with trophoblast dysfunction.

### Supplementary Information

**Additional file 1:** **Figure 1.**-Source data 1. Excel file for Figure 1A and B.

**Additional file 2:** **Figure 1.**-Source data 2. Excel file for Figure 1C.

**Additional file 3:** **Figure 1.**-Source data 3. Excel file for Figure 1D.

**Additional file 4:** **Figure 1.**-Source data 4. Excel file for Figure 1E.

**Additional file 5:** **Figure 1.**-Source data 5. Prism file for Figure 1F-I.

**Additional file 6:** **Figure 1.**-Source data 6. Original image data for Figure 1G and I.

**Additional file 7:** **Figure 2.**-Source data 1. Prism file for Figure 2A-F.

**Additional file 8:** **Figure 2.**-Source data 2. Original image data for Figure 2B.

**Additional file 9:** **Figure 3.**-Source data 1. Prism file for Figure 3B-I.

**Additional file 10:** **Figure 3.**-Source data 2. Original image data for Figure 3A, C, F and I.

**Additional file 11:** **Figure 4.**-Source data 1. Original image data for Figure 4A.

**Additional file 12:** **Figure 4.**-Source data 2. Prism file for Figure 4B-F.

**Additional file 13:** **Figure 4.**-Source data 3. Original image data for Figure 4D and G.

**Additional file 14:** **Figure 5.**-Source data 1. Prism file for Figure 5B-F.

**Additional file 15: Figure 5.**-Source data 2. Original image data for Figure 5C.

## Data Availability

All data generated or analyzed during this study are included in this article and its supplementary information files. Further information and requests for resources and reagents should be directed to and will be fulfilled by Yan Li (ubcliyan@sdu.edu.cn).

## Abbreviations

EVTs: extravillous trophoblasts
siRNA: small interfering RNA
si-*Fn1*: siRNA targeting fibronectin 1
*FN1*: fibronectin 1
ALK4: activin receptor-like kinase
CK8: cytokeratin 8
TGF-β: transforming growth factor-β
FSH: follicle stimulating hormone
ActRI: activin type I receptor
VEGF-A: vascular endothelial growth factor-A
ECM: extracellular matrix
DMEM/F12: Dulbecco’s modified Eagle medium nutrient mixture F-12 Ham
FBS: fetal bovine serum
h: hours
PBS: phosphate-buffered saline
HLA-G: human leukocyte antigen G
CK7: cytokeratin 7
RNA-seq: RNA-sequencing
SRA: Sequence Read Archive
DEGs: differentially expressed genes
GO: Gene Ontology
si-*FN1*: siRNA targeting human *FN1*
CCK-8: cell counting kit-8
EdU: 5-ethynyl-2′-deoxyuridine
RT–qPCR: reverse transcription quantitative real-time PCR
GAPDH: glyceraldehyde-3-phosphate dehydrogenase
BSA: bovine serum albumin
PFA: paraformaldehyde
D1: day 1
IS: implantation sites
IHC: immunohistochemistry
IF: immunofluorescence
ANOVA: analysis of variance
Ctrl: control
si-*ALK4*: siRNA targeting *ALK4*
si-*SMAD4*: siRNA targeting *SMAD4*
DMSO: dimethylsulfoxide
NC: control group

## References

[1] Velicky P, Knöfler M, Pollheimer J (2016). Function and control of human invasive trophoblast subtypes: intrinsic vs. maternal control. Cell Adhes Migr.

[2] Illsley NP, DaSilva-Arnold SC, Zamudio S, Alvarez M, Al-Khan A (2020). Trophoblast invasion: lessons from abnormally invasive placenta (placenta accreta). Placenta.

[3] Tang L, He G, Liu X, Xu W (2017). Progress in the understanding of the etiology and predictability of fetal growth restriction. Reproduction.

[4] Chaddha V, Viero S, Huppertz B, Kingdom J (2004). Developmental biology of the placenta and the origins of placental insufficiency. Semin Fetal Neonatal Med.

[5] Dimitriadis E, White CA, Jones RL, Salamonsen LA (2005). Cytokines, chemokines and growth factors in endometrium related to implantation. Hum Reprod Update.

[6] Guzeloglu-Kayisli O, Kayisli UA, Taylor HS (2009). The role of growth factors and cytokines during implantation: endocrine and paracrine interactions. Semin Reprod Med.

[7] Gupta SK, Malhotra SS, Malik A, Verma S, Chaudhary P (2016). Cell signaling pathways involved during invasion and Syncytialization of trophoblast cells. Am J Reprod Immunol.

[8] Huang T, Hinck AP (2016). Production, isolation, and structural analysis of ligands and receptors of the TGF-β superfamily. Methods Mol Biol.

[9] Vale W, Rivier J, Vaughan J, McClintock R, Corrigan A, Woo W, Karr D, Spiess J (1986). Purification and characterization of an FSH releasing protein from porcine ovarian follicular fluid. Nature.

[10] Ling N, Ying S-Y, Ueno N, Shimasaki S, Esch F, Hotta M, Guillemin R (1986). Pituitary FSH is released by a heterodimer of the β-subunits from the two forms of inhibin. Nature.

[11] Daponte A, Deligeoroglou E, Garas A, Pournaras S, Hadjichristodoulou C, Messinis IE (2013). Activin a and follistatin as biomarkers for ectopic pregnancy and missed abortion. Dis Markers.

[12] Rausch ME, Sammel MD, Takacs P, Chung K, Shaunik A, Barnhart KT (2011). Development of a multiple marker test for ectopic pregnancy. Obstet Gynecol.

[13] Prakash A, Laird S, Tuckerman E, Li TC, Ledger WL (2005). Inhibin a and activin a may be used to predict pregnancy outcome in women with recurrent miscarriage. Fertil Steril.

[14] Florio P, Bruni L, Galleri L, Reis FM, Borges LE, Bocchi C, Litta P, De Leo V, Petraglia F (2010). Evaluation of endometrial activin a secretion for prediction of pregnancy after intrauterine insemination. Fertil Steril.

[15] Lewis KA, Gray PC, Blount AL, MacConell LA, Wiater E, Bilezikjian LM, Vale W (2000). Betaglycan binds inhibin and can mediate functional antagonism of activin signalling. Nature.

[16] Massagué J (2000). How cells read TGF-beta signals. Nat Rev Mol Cell Biol.

[17] Bao YL, Tsuchida K, Liu B, Kurisaki A, Matsuzaki T, Sugino H (2005). Synergistic activity of activin a and basic fibroblast growth factor on tyrosine hydroxylase expression through Smad3 and ERK1/ERK2 MAPK signaling pathways. J Endocrinol.

[18] Vallet S, Mukherjee S, Vaghela N, Hideshima T, Fulciniti M, Pozzi S, Santo L, Cirstea D, Patel K, Sohani AR (2010). Activin a promotes multiple myeloma-induced osteolysis and is a promising target for myeloma bone disease. Proc Natl Acad Sci U S A.

[19] Singh AM, Reynolds D, Cliff T, Ohtsuka S, Mattheyses AL, Sun Y, Menendez L, Kulik M, Dalton S (2012). Signaling network crosstalk in human pluripotent cells: a Smad2/3-regulated switch that controls the balance between self-renewal and differentiation. Cell Stem Cell.

[20] Li Y, Klausen C, Cheng JC, Zhu H, Leung PC (2014). Activin a, B, and AB increase human trophoblast cell invasion by up-regulating N-cadherin. J Clin Endocrinol Metab.

[21] Li Y, Klausen C, Zhu H, Leung PC (2015). Activin a increases human trophoblast invasion by inducing SNAIL-mediated MMP2 up-regulation through ALK4. J Clin Endocrinol Metab.

[22] Li Y, Zhu H, Klausen C, Peng B, Leung PC (2015). Vascular endothelial growth factor-a (VEGF-A) mediates Activin A-induced human trophoblast endothelial-like tube formation. Endocrinology.

[23] Mao Y, Schwarzbauer JE (2005). Fibronectin fibrillogenesis, a cell-mediated matrix assembly process. Matrix Biol.

[24] Rostagno A, Williams MJ, Baron M, Campbell ID, Gold LI (1994). Further characterization of the NH2-terminal fibrin-binding site on fibronectin. J Biol Chem.

[25] Peng Z, Hao M, Tong H, Yang H, Huang B, Zhang Z, Luo KQ (2022). The interactions between integrin α(5)β(1) of liver cancer cells and fibronectin of fibroblasts promote tumor growth and angiogenesis. Int J Biol Sci.

[26] Qiao P, Lu ZR (2020). Fibronectin in the tumor microenvironment. Adv Exp Med Biol.

[27] Lala PK, Nandi P, Hadi A, Halari C (2021). A crossroad between placental and tumor biology: what have we learnt?. Placenta.

[28] Krstic J, Deutsch A, Fuchs J, Gauster M, Gorsek Sparovec T, Hiden U, Krappinger JC, Moser G, Pansy K, Szmyra M, et al. (dis) similarities between the Decidual and tumor microenvironment. Biomedicines. 2022:10. 10.3390/biomedicines10051065 PMC9138511 35625802

[29] Fagerberg L, Hallström BM, Oksvold P, Kampf C, Djureinovic D, Odeberg J, Habuka M, Tahmasebpoor S, Danielsson A, Edlund K (2014). Analysis of the human tissue-specific expression by genome-wide integration of transcriptomics and antibody-based proteomics. Mol Cell Proteomics.

[30] Uhlén M, Fagerberg L, Hallström BM, Lindskog C, Oksvold P, Mardinoglu A, Sivertsson Å, Kampf C, Sjöstedt E, Asplund A (2015). Proteomics. Tissue-based map of the human proteome. Science.

[31] Zhu S, Li Z, Cui L, Ban Y, Leung PCK, Li Y, Ma J (2021). Activin a increases human trophoblast invasion by upregulating integrin β1 through ALK4. FASEB J.

[32] Eriksen HO, Hansen PK, Brocks V, Jensen BA (1987). Plasma fibronectin concentration in normal pregnancy and pre-eclampsia. Acta Obstet Gynecol Scand.

[33] Chavarría ME, Lara-González L, González-Gleason A, Sojo I, Reyes A (2002). Maternal plasma cellular fibronectin concentrations in normal and preeclamptic pregnancies: a longitudinal study for early prediction of preeclampsia. Am J Obstet Gynecol.

[34] Fowler PA, Evans LW, Groome NP, Templeton A, Knight PG (1998). A longitudinal study of maternal serum inhibin-a, inhibin-B, activin-a, activin-AB, pro-alphaC and follistatin during pregnancy. Hum Reprod.

[35] Schneider-Kolsky M, D'Antona D, Evans LW, Taylor N, O'Connor A, Groome NP, de Kretser D, Wallace EM (2000). Maternal serum total activin a and follistatin in pregnancy and parturition. Bjog.

[36] Ji J, Chen L, Zhuang Y, Han Y, Tang W, Xia F (2020). Fibronectin 1 inhibits the apoptosis of human trophoblasts by activating the PI3K/Akt signaling pathway. Int J Mol Med.

[37] Irving JA, Lysiak JJ, Graham CH, Hearn S, Han VK, Lala PK (1995). Characteristics of trophoblast cells migrating from first trimester chorionic villus explants and propagated in culture. Placenta.

[38] Love MI, Huber W, Anders S (2014). Moderated estimation of fold change and dispersion for RNA-seq data with DESeq2. Genome Biol.

[39] Blighe K, Rana S, Lewis M (2023). EnhancedVolcano: publication-ready volcano plots with enhanced colouri ng and labeling.

[40] Bardou P, Mariette J, Escudié F, Djemiel C, Klopp C (2014). jvenn: an interactive Venn diagram viewer. BMC Bioinformatics.

[41] Kumar L, Futschik M. Mfuzz: a software package for soft clustering of microarray data. Bioinformation. 2007:5–7. 10.6026/97320630002005 PMC2139991 18084642

[42] Futschik M, Carlisle B. Noise robust clustering of gene expression time-course data. J Bioinforma Comput Biol. 2005:965–88. 10.1142/s0219720005001375 16078370

[43] Wu T, Hu E, Xu S, Chen M, Guo P, Dai Z, Feng T, Zhou L, Tang W, Zhan L (2021). ClusterProfiler 4.0: A universal enrichment tool for interpreting omic s data. The Innovation.

[44] Team RC (2023). R: a language and environment for statistical computing.

[45] Yu L, Li D, Liao QP, Yang HX, Cao B, Fu G, Ye G, Bai Y, Wang H, Cui N (2012). High levels of activin a detected in preeclamptic placenta induce trophoblast cell apoptosis by promoting nodal signaling. J Clin Endocrinol Metab.

[46] Muttukrishna S, Jauniaux E, McGarrigle H, Groome N, Rodeck CH (2004). In-vivo concentrations of inhibins, activin a and follistatin in human early pregnancy. Reprod BioMed Online.

[47] Lobo SE, Leonel LC, Miranda CM, Coelho TM, Ferreira GA, Mess A, Abrão MS, Miglino MA (2016). The placenta as an organ and a source of stem cells and extracellular matrix: a review. Cells Tissues Organs.

[48] Singh P, Carraher C, Schwarzbauer JE (2010). Assembly of fibronectin extracellular matrix. Annu Rev Cell Dev Biol.

[49] Akerman ME, Pilch J, Peters D, Ruoslahti E (2005). Angiostatic peptides use plasma fibronectin to home to angiogenic vasculature. Proc Natl Acad Sci U S A.

[50] Knowles LM, Malik G, Hood BL, Conrads TP, Pilch J (2012). CLT1 targets angiogenic endothelium through CLIC1 and fibronectin. Angiogenesis.

[51] Ruoslahti E (1988). Fibronectin and its receptors. Annu Rev Biochem.

[52] Tamminen JA, Yin M, Rönty M, Sutinen E, Pasternack A, Ritvos O, Myllärniemi M, Koli K (2015). Overexpression of activin-a and -B in malignant mesothelioma - attenuated Smad3 signaling responses and ERK activation promote cell migration and invasive growth. Exp Cell Res.

[53] Haider S, Lackner AI, Dietrich B, Kunihs V, Haslinger P, Meinhardt G, Maxian T, Saleh L, Fiala C, Pollheimer J (2022). Transforming growth factor-β signaling governs the differentiation program of extravillous trophoblasts in the developing human placenta. Proc Natl Acad Sci U S A.

[54] Li Y, Yan J, Chang HM, Chen ZJ, Leung PCK (2021). Roles of TGF-β superfamily proteins in Extravillous trophoblast invasion. Trends Endocrinol Metab.

[55] Xu RH, Chen X, Li DS, Li R, Addicks GC, Glennon C, Zwaka TP, Thomson JA (2002). BMP4 initiates human embryonic stem cell differentiation to trophoblast. Nat Biotechnol.

[56] Osnato A, Brown S, Krueger C, Andrews S, Collier AJ, Nakanoh S, Quiroga Londoño M, Wesley BT, Muraro D, Brumm AS, et al. TGFβ signalling is required to maintain pluripotency of human naïve pluripotent stem cells. Elife. 2021;10 10.7554/eLife.67259 PMC8410071 34463252

[57] Zhao HJ, Klausen C, Zhu H, Chang HM, Li Y, Leung PCK (2020). Bone morphogenetic protein 2 promotes human trophoblast cell invasion and endothelial-like tube formation through ID1-mediated upregulation of IGF binding protein-3. FASEB J.

[58] Xie J, Zhu H, Chang HM, Klausen C, Dong M, Leung PCK (2020). GDF8 promotes the cell invasiveness in human trophoblasts by upregulating the expression of Follistatin-like 3 through the ALK5-SMAD2/3 signaling pathway. Front Cell Dev Biol.

[59] Zhao HJ, Klausen C, Li Y, Zhu H, Wang YL, Leung PCK (2018). Bone morphogenetic protein 2 promotes human trophoblast cell invasion by upregulating N-cadherin via non-canonical SMAD2/3 signaling. Cell Death Dis.

[60] Sun F, Cheng L, Guo L, Su S, Li Y, Yan J (2022). Activin a promotes human trophoblast invasion by upregulating integrin β3 via ALK4-SMAD4 signaling. Placenta.

[61] Brosens I, Puttemans P, Benagiano G (2019). Placental bed research: I. The placental bed: from spiral arteries remodeling to the great obstetrical syndromes. Am J Obstet Gynecol.

[62] Pijnenborg R, Vercruysse L, Hanssens M (2006). The uterine spiral arteries in human pregnancy: facts and controversies. Placenta.

[63] Li Y, Cheng Z, Yu F, Zhang Q, Yu S, Ding F, He Q (2022). Activin a secreted from peripheral nerve fibroblasts promotes proliferation and migration of Schwann cells. Front Mol Neurosci.

[64] Daitoku N, Miyamoto Y, Hiyoshi Y, Tokunaga R, Sakamoto Y, Sawayama H, Ishimoto T, Baba Y, Yoshida N, Baba H. Activin a promotes cell proliferation, invasion and migration and predicts poor prognosis in patients with colorectal cancer. Oncol Rep. 2022;47 10.3892/or.2022.8318 PMC9073419 35445735

[65] Aldo PB, Krikun G, Visintin I, Lockwood C, Romero R, Mor G (2007). A novel three-dimensional in vitro system to study trophoblast-endothelium cell interactions. Am J Reprod Immunol.

[66] Zhong C, Tao B, Tang F, Yang X, Peng T, You J, Xia K, Xia X, Chen L, Peng L (2021). Remodeling cancer stemness by collagen/fibronectin via the AKT and CDC42 signaling pathway crosstalk in glioma. Theranostics.

[67] Lenselink EA (2015). Role of fibronectin in normal wound healing. Int Wound J.

[68] Reichsoellner M, Raggam RB, Wagner J, Krause R, Hoenigl M (2014). Clinical evaluation of multiple inflammation biomarkers for diagnosis and prognosis for patients with systemic inflammatory response syndrome. J Clin Microbiol.

[69] Date M, Matsuzaki K, Matsushita M, Tahashi Y, Sakitani K, Inoue K (2000). Differential regulation of activin a for hepatocyte growth and fibronectin synthesis in rat liver injury. J Hepatol.

[70] Islam MS, Ciavattini A, Petraglia F, Castellucci M, Ciarmela P (2018). Extracellular matrix in uterine leiomyoma pathogenesis: a potential target for future therapeutics. Hum Reprod Update.

[71] Islam MS, Catherino WH, Protic O, Janjusevic M, Gray PC, Giannubilo SR, Ciavattini A, Lamanna P, Tranquilli AL, Petraglia F (2014). Role of activin-a and myostatin and their signaling pathway in human myometrial and leiomyoma cell function. J Clin Endocrinol Metab.

[72] Berghella V, Saccone G (2019). Fetal fibronectin testing for reducing the risk of preterm birth. Cochrane Database Syst Rev.

[73] Michelsen TM, Henriksen T, Reinhold D, Powell TL, Jansson T (2019). The human placental proteome secreted into the maternal and fetal circulations in normal pregnancy based on 4-vessel sampling. FASEB J.

[74] George EL, Georges-Labouesse EN, Patel-King RS, Rayburn H, Hynes RO (1993). Defects in mesoderm, neural tube and vascular development in mouse embryos lacking fibronectin. Development.

[75] Bucher K, Rodríguez-Bocanegra E, Dauletbekov D, Fischer MD (2021). Immune responses to retinal gene therapy using adeno-associated viral vectors - implications for treatment success and safety. Prog Retin Eye Res.

[76] Li JX, Wu SP, Guo XL, Tang R, Huang BY, Chen XQ, Chen Y, Hou LH, Liu JX, Zhong J (2022). Safety and immunogenicity of heterologous boost immunisation with an orally administered aerosolised Ad5-nCoV after two-dose priming with an inactivated SARS-CoV-2 vaccine in Chinese adults: a randomised, open-label, single-Centre trial. Lancet Respir Med.

[77] Sung DC, Chen X, Chen M, Yang J, Schultz S, Babu A, Xu Y, Gao S, Keller TS IV, Mericko-Ishizuka P, Lee M, et al. VE-cadherin enables trophoblast endovascular invasion and spiral artery remodeling during placental development. Elife. 2022;11 10.7554/eLife.77241 PMC9106330 35486098

[78] Pringle KG, Roberts CT (2007). New light on early post-implantation pregnancy in the mouse: roles for insulin-like growth factor-II (IGF-II)?. Placenta.

[79] Whitley GS, Cartwright JE (2010). Cellular and molecular regulation of spiral artery remodelling: lessons from the cardiovascular field. Placenta.

