# Supplementary figures and images for: Fibronectin mediates activin A-promoted human trophoblast migration and acquisition of endothelial-like phenotype
Source: Cell Commun Signal. 2024 Jan 23;22:61. doi: 10.1186/s12964-023-01463-z (PMC10807102; doi:10.1186/s12964-023-01463-z)

Figure 1G

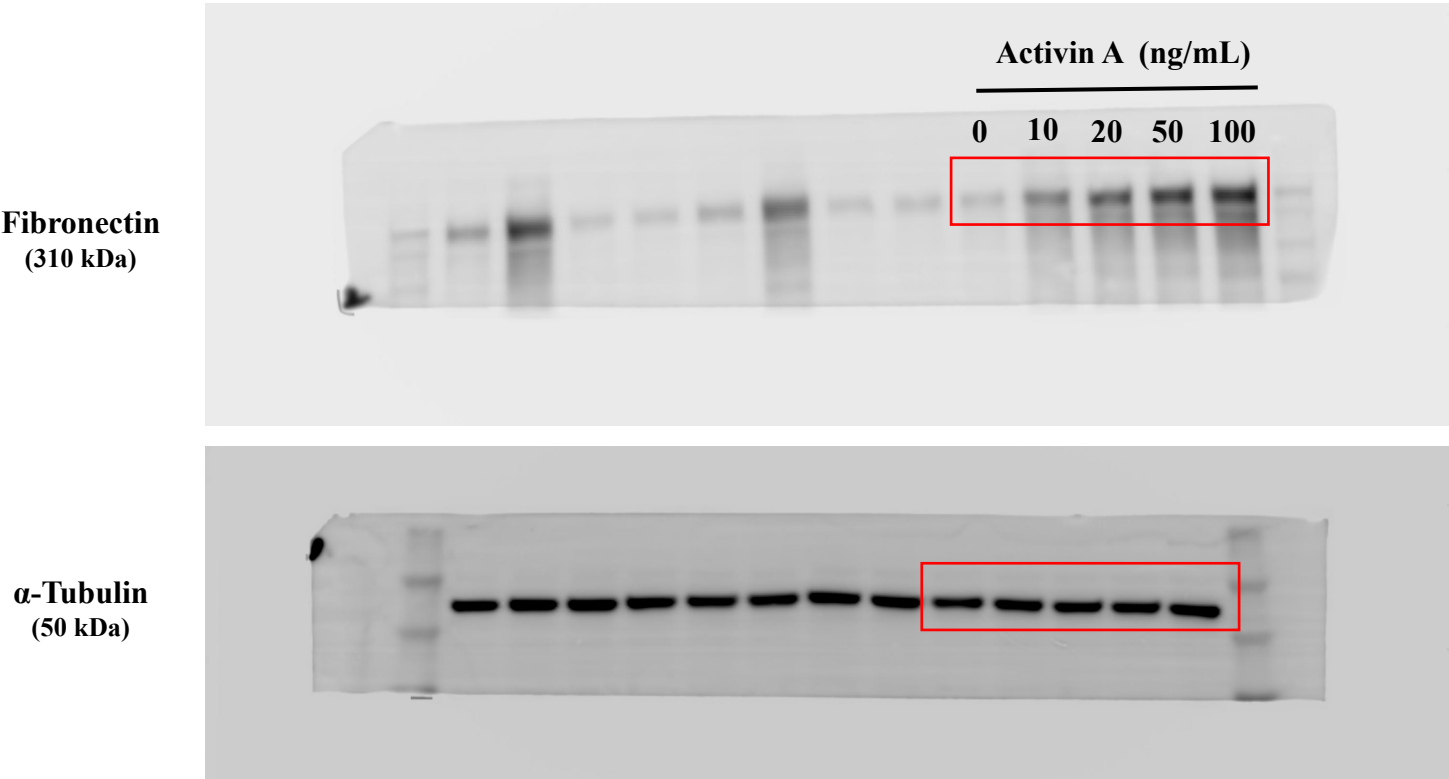

Figure 1I

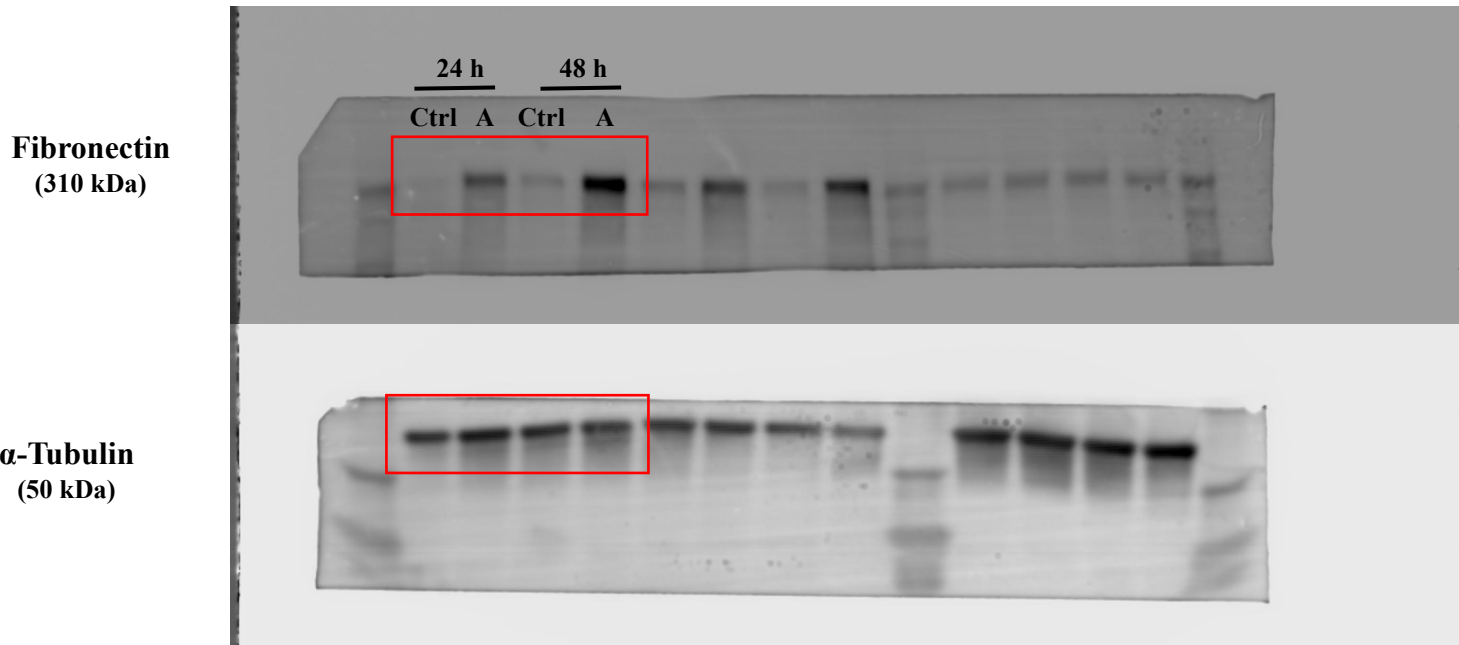

Supplement: Supplementary file 6 — Additional file 6: Figure 1.-Source data 6. Original image data for Figure 1G and I. [file 12964_2023_1463_MOESM6_ESM.pdf]

Figure 2B

Fibronectin  
(310 kDa)

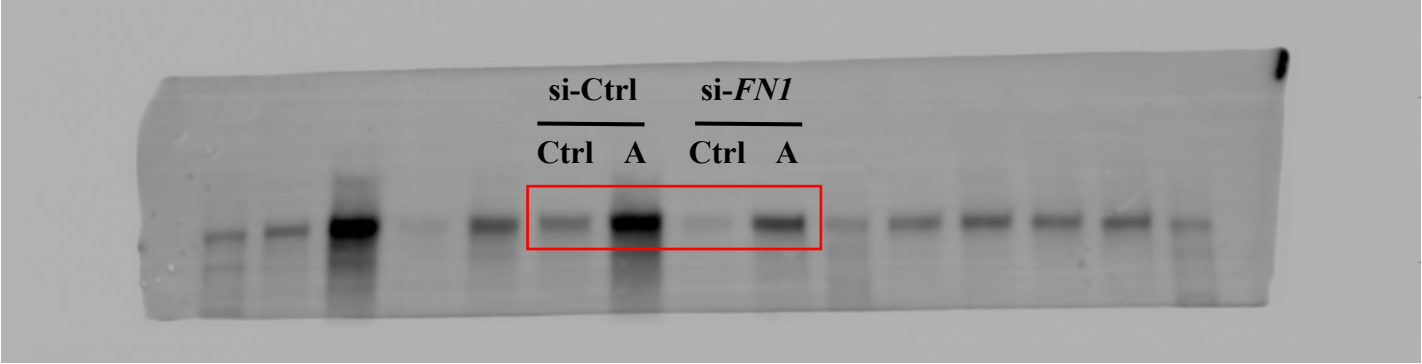

$\alpha$ -Tubulin  
(50 kDa)

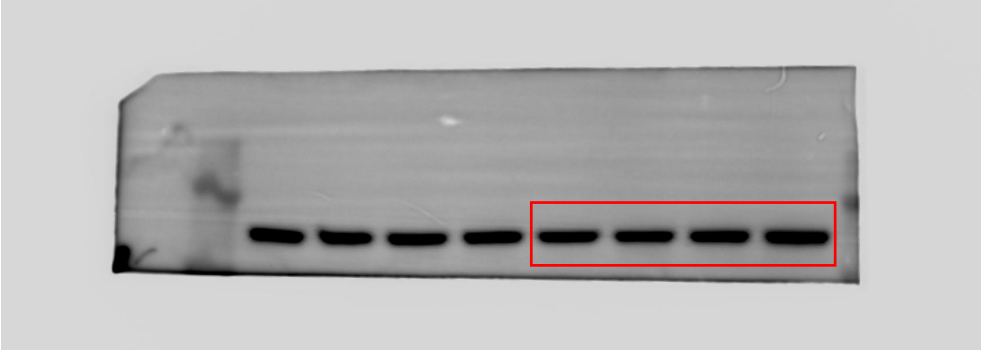

Supplement: Supplementary file 8 — Additional file 8: Figure 2.-Source data 2. Original image data for Figure 2B. [file 12964_2023_1463_MOESM8_ESM.pdf]

**P-SMAD2**  
**(60 kDa)**

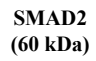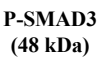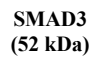

**Fibronectin**  
(310 kDa)

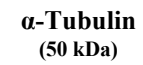

Figure 3F

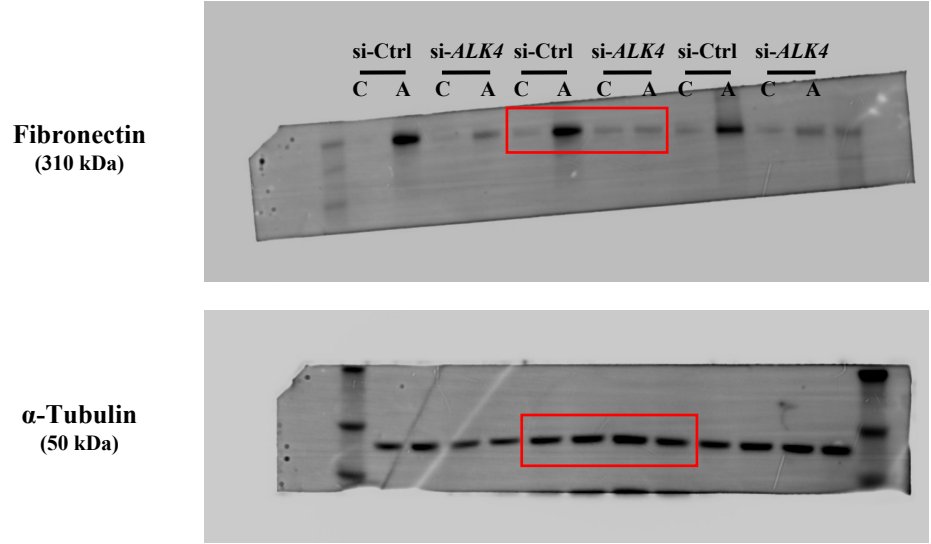

Figure 3I

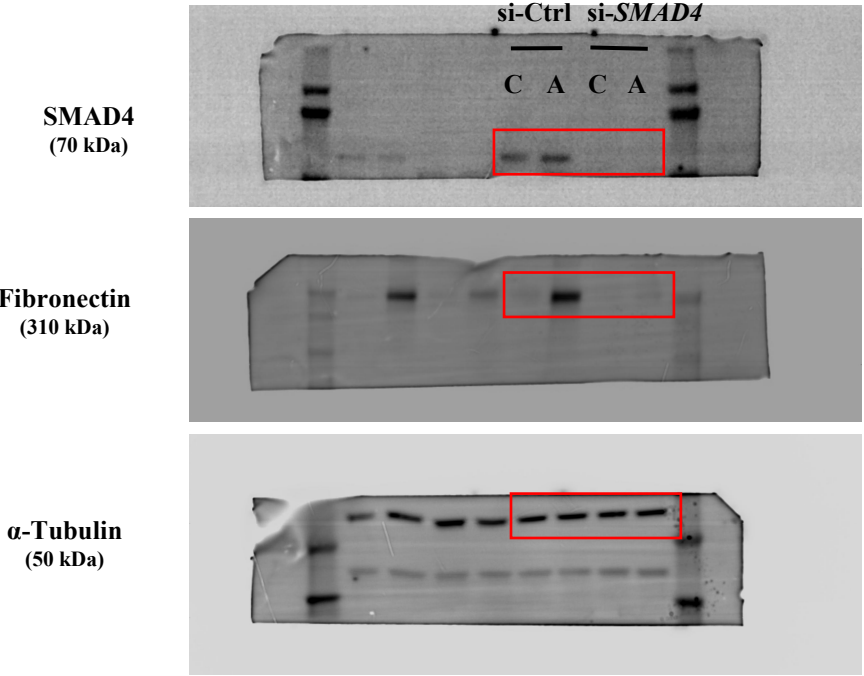

Supplement: Supplementary file 10 — Additional file 10: Figure 3.-Source data 2. Original image data for Figure 3A, C, F and I. [file 12964_2023_1463_MOESM10_ESM.pdf]

Figure 4A

Hoechst

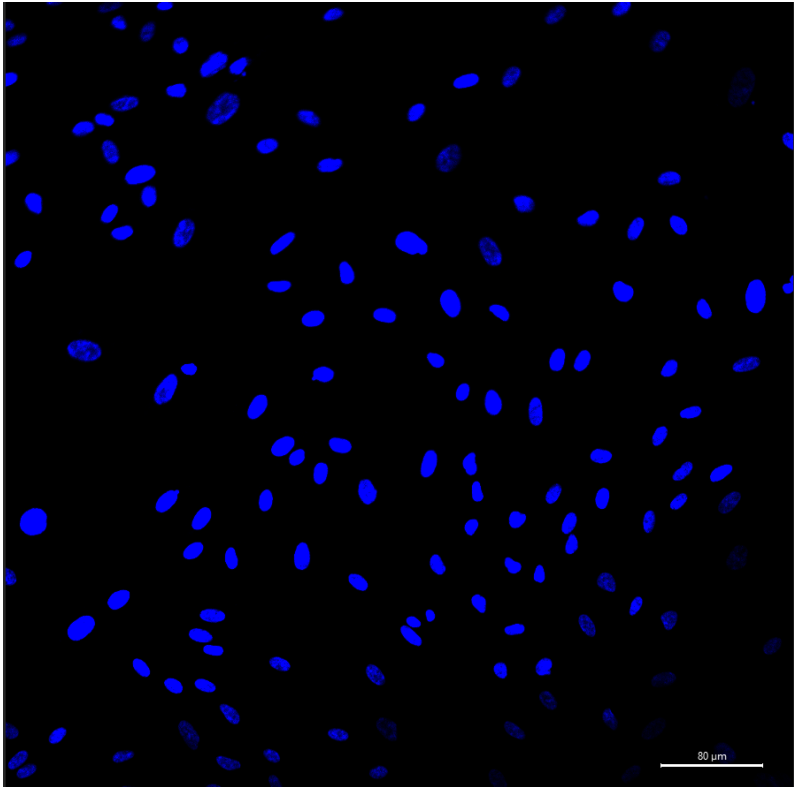

Cytokeratin 7/HLA-G/Hoechst

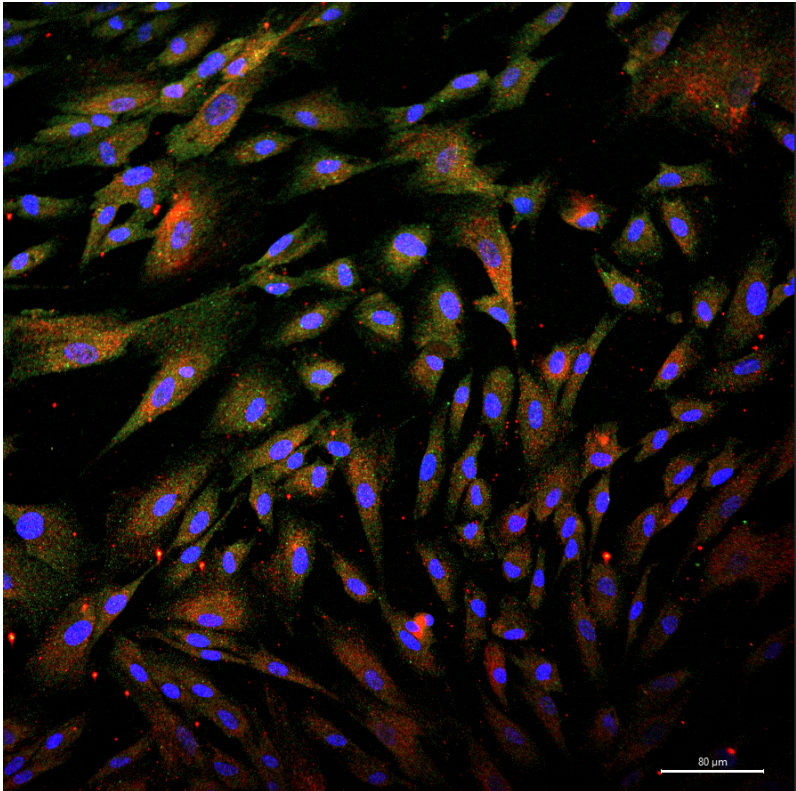

Cytokeratin 7/Hoechst

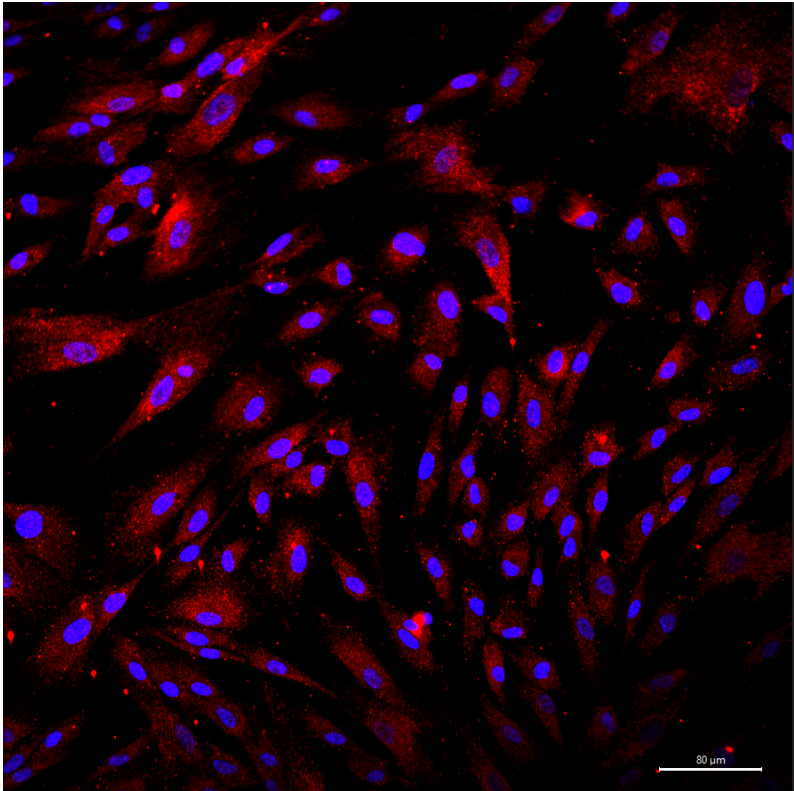

HLA-G/Hoechst

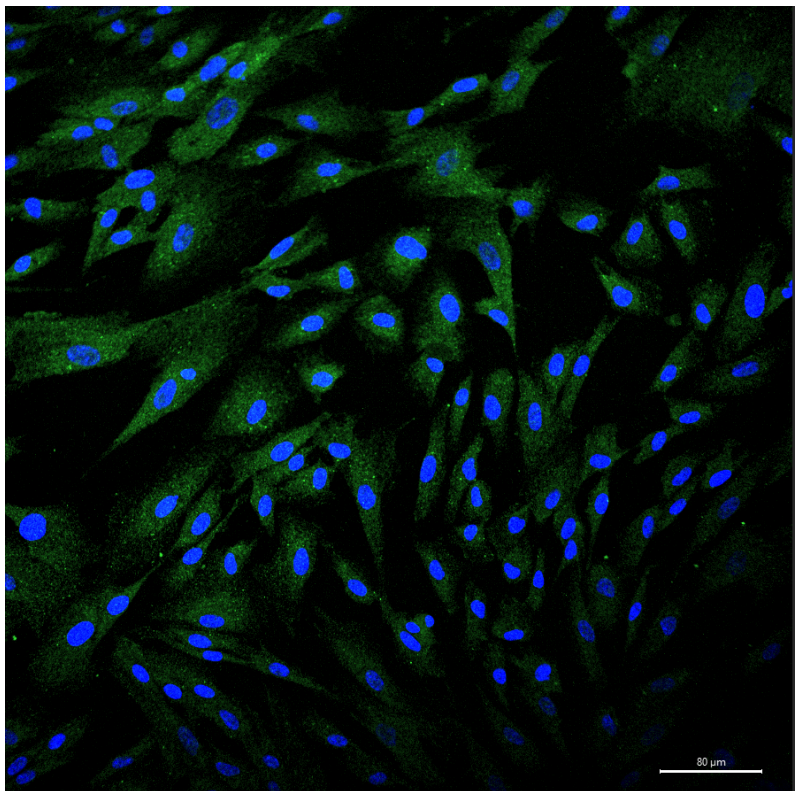

Supplement: Supplementary file 11 — Additional file 11: Figure 4.-Source data 1. Original image data for Figure 4A. [file 12964_2023_1463_MOESM11_ESM.pdf]

Figure 5C

**Fibronectin**  
**(310 kDa)**

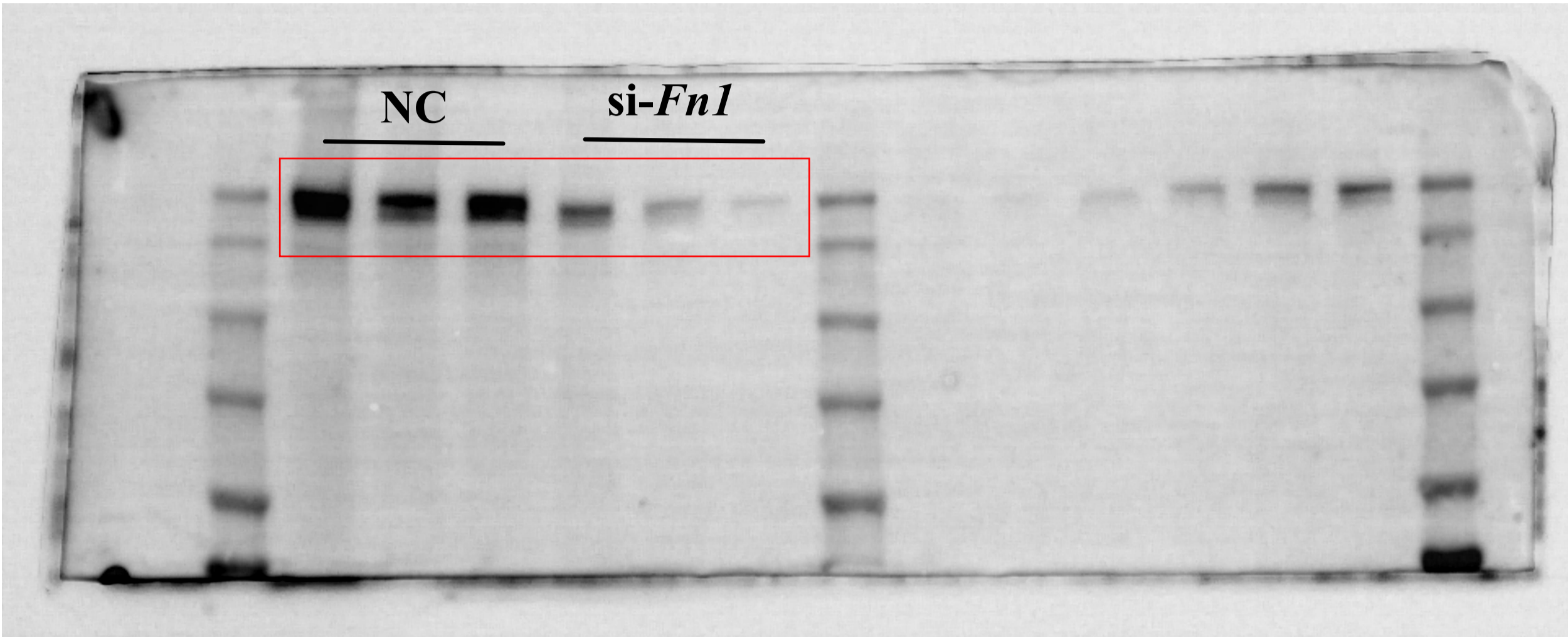

**$\alpha$ -Tubulin**  
**(50 kDa)**

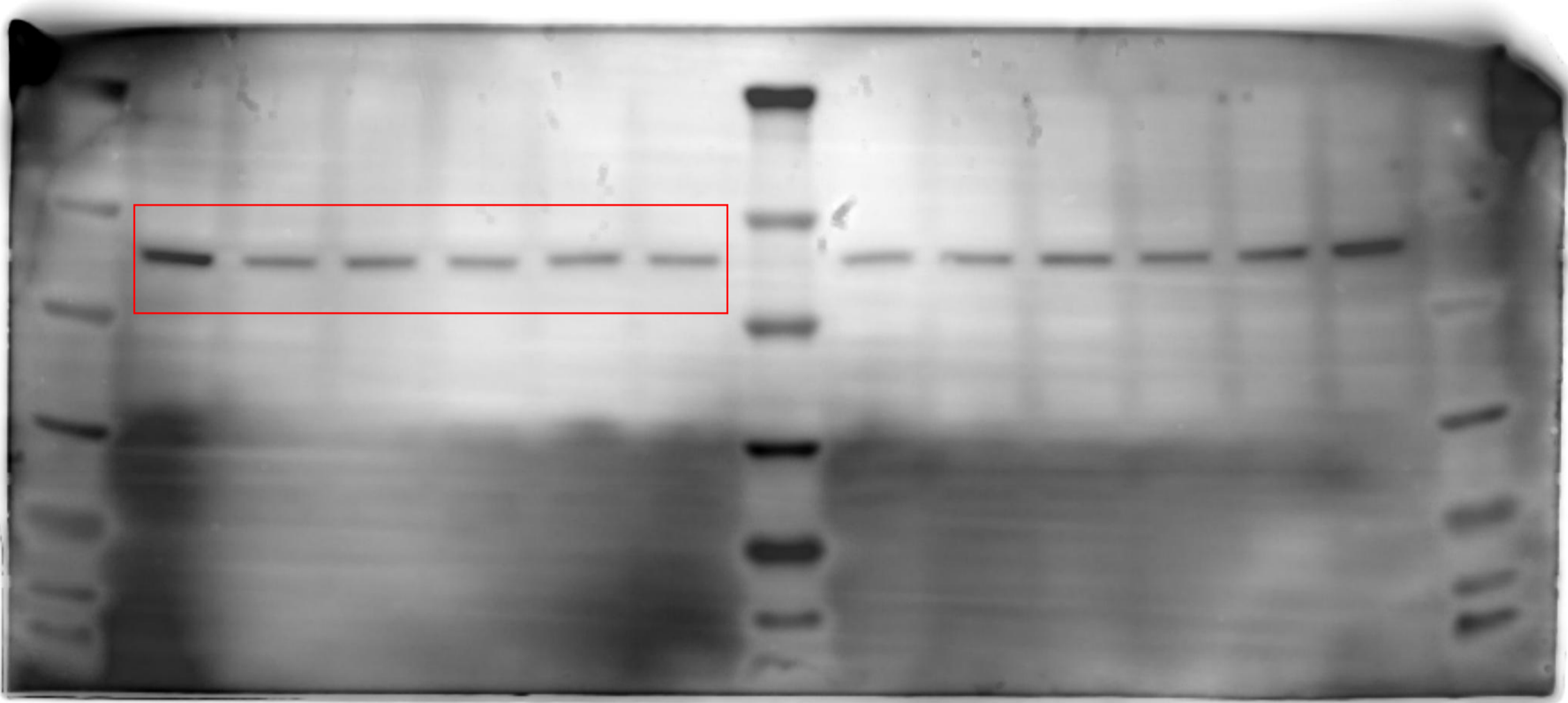

Supplement: Supplementary file 15 — Additional file 15: Figure 5.-Source data 2. Original image data for Figure 5C. [file 12964_2023_1463_MOESM15_ESM.pdf]
