# Supplementary material for: Fibronectin mediates activin A-promoted human trophoblast migration and acquisition of endothelial-like phenotype
Source: Cell Commun Signal. 2024 Jan 23;22:61. doi: 10.1186/s12964-023-01463-z (PMC10807102; doi:10.1186/s12964-023-01463-z)

Figure 4D

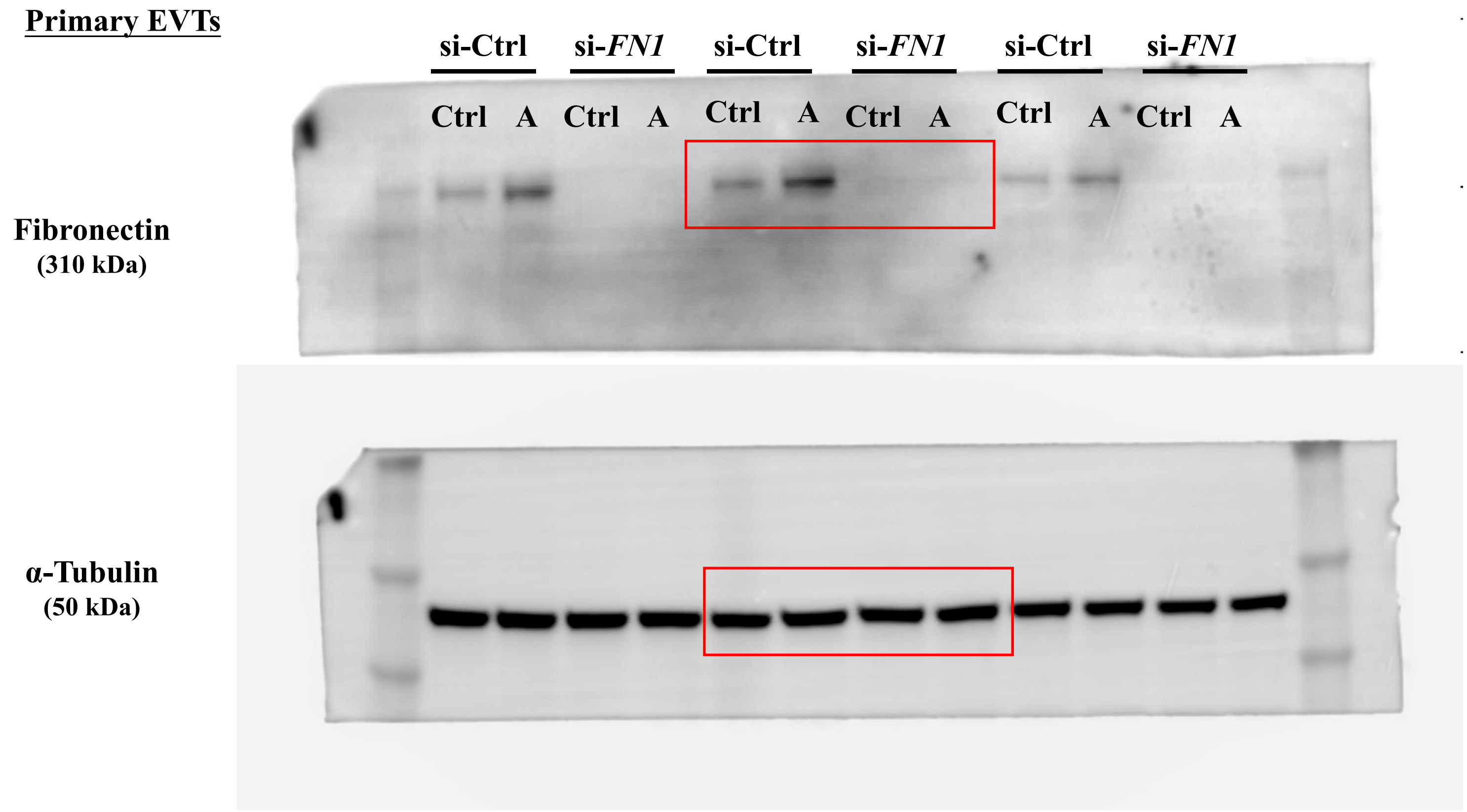

Figure 4G

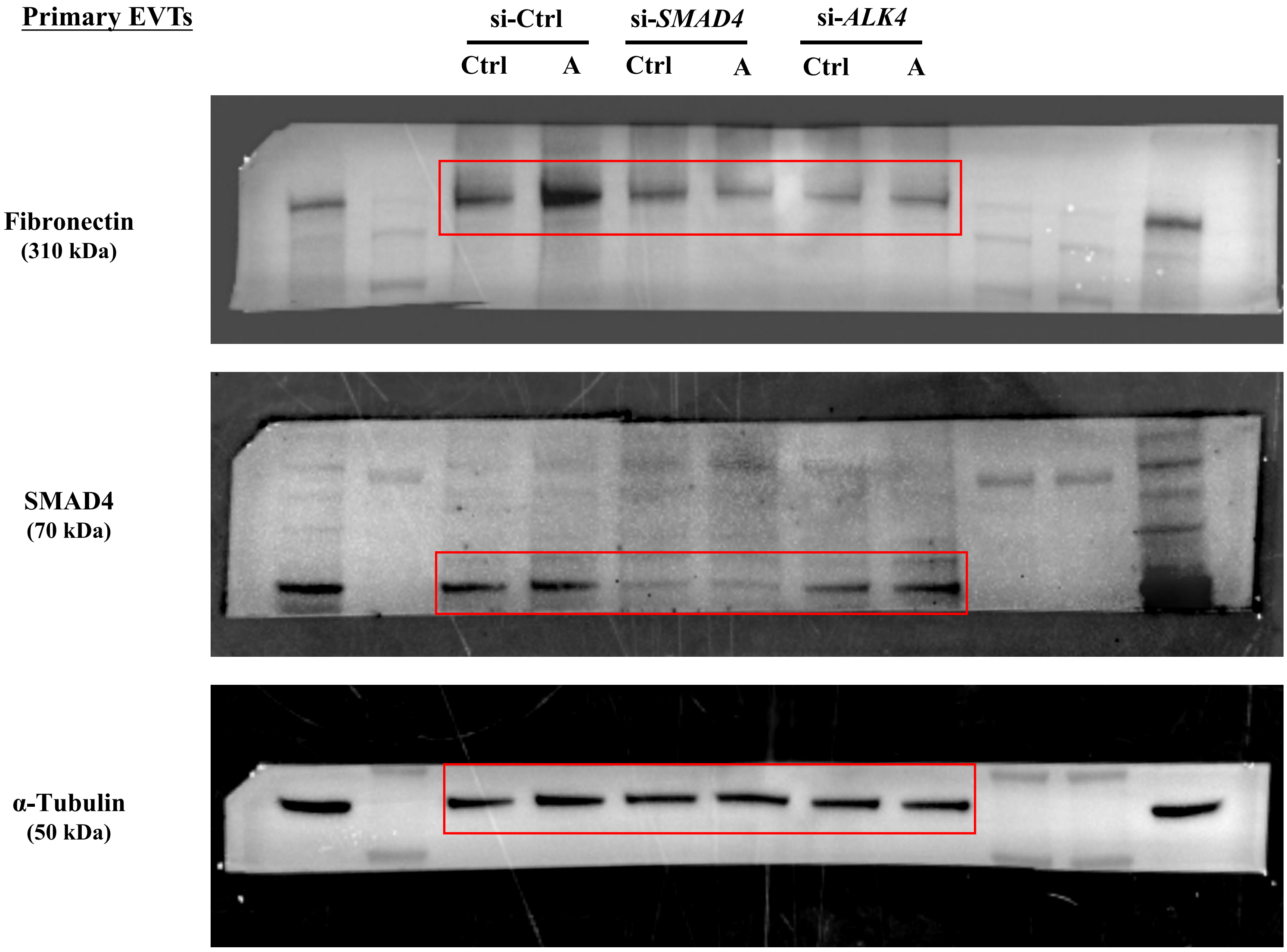

Supplement: Supplementary file 13 — Additional file 13: Figure 4.-Source data 3. Original image data for Figure 4D and G. [file 12964_2023_1463_MOESM13_ESM.pdf]
